# Energetics and Kinetics of Hydrogen Electrosorption on a Graphene-Covered Pt(111) Electrode

**DOI:** 10.1021/jacsau.2c00648

**Published:** 2023-01-18

**Authors:** Nakkiran Arulmozhi, Selwyn Hanselman, Viorica Tudor, Xiaoting Chen, David van Velden, Grégory F. Schneider, Federico Calle-Vallejo, Marc T. M. Koper

**Affiliations:** † Leiden Institute of Chemistry, Leiden University, P.O. Box 9502, Leiden 2300 RA, The Netherlands; ‡ Department of Materials Science and Chemical Physics & Institute of Theoretical and Computational Chemistry (IQTCUB), University of Barcelona, Martí i Franquès 1, 08028 Barcelona, Spain; § Nano-Bio Spectroscopy Group and European Theoretical Spectroscopy Facility (ETSF), Department of Polymers and Advanced Materials: Physics, Chemistry and Technology, University of the Basque Country UPV/EHU, Av. Tolosa 72, 20018 San Sebastián, Spain; ⊥ IKERBASQUE, Basque Foundation for Science, Plaza de Euskadi 5, 48009 Bilbao, Spain

**Keywords:** Pt(111), electroadsorption, graphene, proton permeation, surface−membrane interaction

## Abstract

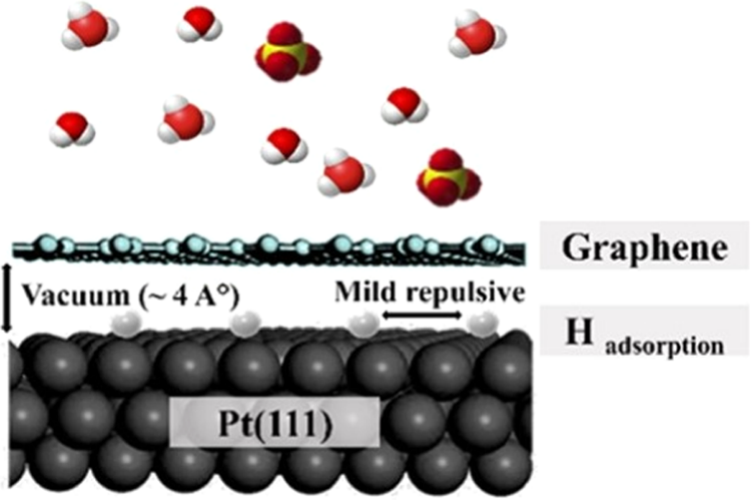

The Angstrom-scale space between graphene and its substrate provides an attractive playground for scientific exploration and can lead to breakthrough applications. Here, we report the energetics and kinetics of hydrogen electrosorption on a graphene-covered Pt(111) electrode using electrochemical experiments, in situ spectroscopy, and density functional theory calculations. The graphene overlayer influences the hydrogen adsorption on Pt(111) by shielding the ions from the interface and weakening the Pt–H bond energy. Analysis of the proton permeation resistance with controlled graphene defect density proves that the domain boundary defects and point defects are the pathways for proton permeation in the graphene layer, in agreement with density functional theory (DFT) calculations of the lowest energy proton permeation pathways. Although graphene blocks the interaction of anions with the Pt(111) surfaces, anions do adsorb near the defects: the rate constant for hydrogen permeation is sensitively dependent on anion identity and concentration.

## Introduction

An electrode covered by a two-dimensional (2D) membrane is a novel and attractive design approach^1−8^ for improving the performance and the lifetime of electrochemical systems for catalysis,^1,2^ batteries,^4^ CO_2_ valorization,^5^ biosensors,^6^ and other electrochemical devices.^8^ The 2D overlayer on the electrode supplies a confined space^9^ (i.e., the Angstrom-scale separation between the 2D layer and the metal substrate) that can act as a stable nanoreactor for molecular adsorption and chemical reactions.^9−11^ In addition, the graphene monolayer can be engineered to permeate the required ions and molecules to the 2D space while blocking other undesirable species likely to degrade or compromise the electrode’s function.^8^ Therefore, a fundamental-level investigation of electroadsorption on a metal electrode covered by a 2D material is essential for scientific and applied purposes.

A model system to investigate is the electroadsorption of hydrogen in the Angstrom-scale space between the graphene layer and the Pt(111) electrode (denoted G-Pt(111)). On the one hand, the methodology for growing graphene over Pt has been optimized,^12^ and the interaction of graphene with Pt(111) has been explored using theoretical^13^ and experimental tools,^14^ and the gap between graphene and Pt(111) is established to be 3.7 Å.^15^ On the other hand, the Pt(111)/electrolyte system has been studied for more than four decades and displays “fingerprint” voltammetric profiles for many processes, such as hydrogen adsorption, anion adsorption, order/disorder phase transitions, and surface oxidation.^16^ Recently, Fu et al.^17^ demonstrated that for a G-Pt(111) electrode, the proton selectively transports via graphene and deposits onto Pt(111). In contrast, anions are prohibited from binding to the Pt(111) surface. Our group has recently shown that sufficiently defective graphene on Pt(111) may in fact improve the catalytic activity for hydrogen evolution.^18^

This paper aims to investigate the nature of a G-Pt(111)/electrolyte interface and the fundamental aspects of the electroadsorbed hydrogen in the 2D space. First, by temperature-dependent electrochemical experiments, we evaluate the thermodynamic state functions, lateral interaction energy, as well as the Pt(111)–H surface bond energy (*E*_Pt(111)–H_). The lateral repulsion and the Pt(111)–H surface bond energy are compared to the adsorption of hydrogen on the unmodified Pt(111) electrode and to corresponding density functional theory (DFT) calculations. Next, the mechanism of proton permeation through graphene is investigated via in situ Raman spectroscopy and impedance spectroscopy. The potential-dependent Raman spectra show no evidence of proton tunneling via graphene hydrogenation. From the impedance and Raman spectra of proton permeation as a function of the graphene defect density, we conclude that proton permeation occurs via defects. We will also show that although anions do not bind to the graphene-modified Pt(111) surface, they impact the kinetics of proton permeation, presumably by adsorbing at or near the defects in the graphene overlayer.

## Results and Discussion

### Hydrogen Electroadsorption on G-Pt(111)

**Figure 1:**
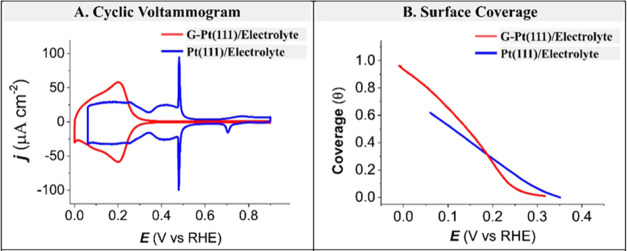
Comparison of hydrogen adsorption/desorption of G-Pt(111) (red) and its energetics in comparison to bare Pt(111) electrode. (A) Cyclic voltammogram profiles in 0.00–0.90 V range for G-Pt(111), bare Pt(111) in 0.1 M aqueous H_2_SO_4_. (B) Surface coverage profile with respect to electrode potential in 0.1 M aqueous H_2_SO_4_.

Figure 1A shows the cyclic voltammograms (CVs) for bare Pt(111) and G-Pt(111) (blue and red curves, respectively) as obtained in 0.1 M H_2_SO_4_ electrolyte solutions. From these data, H binding energies and H surface coverages can be determined.^19−21^Figure 1B shows the hydrogen coverage as a function of the applied potential, obtained via current integration (the hydrogen coverage on Pt(111) actually increases until ca. 1 ML at −0.1 V, as shown by Strmcnik et al. using transient measurements^22^). Several noticeable features can be identified in the CV profile of G-Pt(111). Compared to the bare Pt(111) electrode, the voltammetric region attributed to the bisulfate adsorption/desorption (∼0.35 to 0.50 V vs reversible hydrogen electrode (RHE)) and the sharp spikes of the anion adlayer order/disorder phase transition (∼0.45 V) have disappeared. This is because graphene blocks the interaction of all ions and molecules to the Pt(111), except for hydrogen adsorption.^17,23^ From Figure 1B, it is observed that the onset potential of hydrogen adsorption on the G-Pt(111) electrode (red curve) has slightly shifted to more negative potentials compared to the bare Pt(111) electrode (blue curve). This shift suggests that the binding of H adsorption on Pt(111) is weakened due to the presence of graphene. The overall CV and isotherm shape of hydrogen adsorption on the G-Pt(111) electrode, compared to Pt(111) electrode, is narrower and steeper, suggesting a weakening in the repulsive interactions between the adsorbed hydrogen atoms.^24,25^ In addition, the onset of hydrogen evolution on G-Pt(111) has shifted to more negative potentials, close to 0.0 V^18^ (compared to ∼0.1 V for Pt(111)^16,18^). G-Pt(111) reaches the full H-monolayer (Figure 1B) before hydrogen evolution (compared to hydrogen evolution at ∼65% H-coverage for Pt(111)). The shape of the surface coverage curve in Figure 1B can be correlated to lateral interactions via the Frumkin adsorption isotherm.^24,25^ Overall,the presence of the graphene cover can influence the adsorbed hydrogen–Pt(111) in two ways. One, by shielding the solvent and the electrolyte, namely, water and ionic species, from the interface by the 3.7 Å gap between graphene and Pt(111).^15^ Second, graphene itself can weaken the Pt–H bond energy because the Pt–H bond strength in G/Pt(111) electrode is slightly lower than the bond strength of Pt–H under UHV conditions (see also DFT calculations below).

### Mechanism of Proton Permeation

The mechanism of proton permeation through graphene is still under debate. Geim et al. have suggested that graphene can be partially hydrogenated during the measurements,^23,26^ making its lattice slightly sparser and, thus, more permeable to protons. Others have attributed the observed proton currents to atomic-scale lattice defects, including vacancies.^27^ Raman spectroscopy is sensitive to the hydrogenation of graphene;^28^ therefore, in situ Raman spectra of graphene during proton adsorption can shed light on graphene hydrogenation. In addition, impedance spectroscopy measurements allow us to track the rate of electrochemical adsorption of hydrogen onto the Pt(111) surface via permeation through graphene and how it depends on defect density.

**Figure 2:**
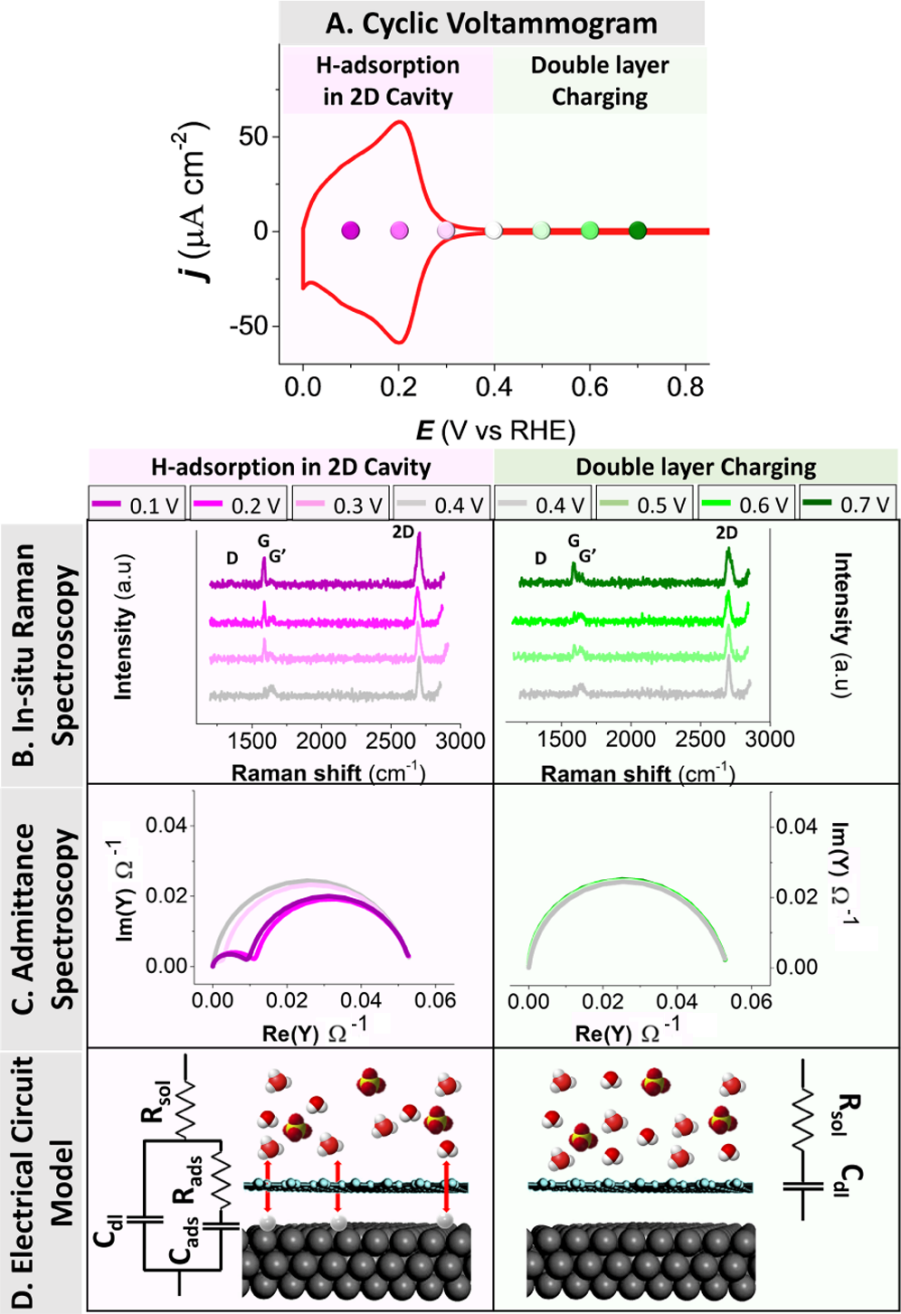
Potential-dependent Raman and admittance analysis. (A) CV profile in the −0.03 to 0.80 V range of G-Pt(111) in 0.1 M aqueous H_2_SO_4_ at room temperature with the potentials indicated for which Raman and admittance spectra were taken. (B) In situ Raman spectra in the 0.10–0.70 V range of G-Pt(111) in 0.1 M aqueous H_2_SO_4_ at room temperature. (C) Admittance spectra in the 0.10–0.70 V range of G-Pt(111) in 0.1 M aqueous H_2_SO_4_ at room temperature. (D) Pictorial representation of electrochemical processes using an equivalent circuit, where *R*_s_ and *R*_ads_ stand for the resistance of the solution and the adsorption resistance, while *C*_dl_ and *C*_ads_ represent the double-layer capacitance and the pseudo-capacitance of adsorbed hydrogen, respectively.

Figure 2 shows the in situ Raman spectra and the electrochemical impedance spectroscopy (EIS) measurements as a function of potential. Figure 2A shows the CV profile of G-Pt(111) in 0.1 M aqueous H_2_SO_4_ with the different potentials probed by in situ Raman spectroscopy and EIS indicated by different colors. Figure 2B,C shows in situ Raman spectra and the admittance (inverse impedance) spectra of G-Pt(111) in 0.1 M aqueous H_2_SO_4_ in the 0.10–0.70 V range, respectively. Figure 2D schematizes the electrochemical processes and the corresponding equivalent electric circuit (EEC) derived from the admittance spectra.

The cyclic voltammogram (Figure 2A) indicates two distinct processes, namely, (i) double-layer charging (0.80–0.40 V) (pink region) and (ii) underpotential deposition of hydrogen onto Pt(111) in the presence of graphene (0.4–0.1 V) (green region). The Raman spectra in Figure 2B do not show the emergence of a D peak, which is a fingerprint for defects or graphene hydrogenation.^28^ Therefore, graphene hydrogenation does not occur to a significant extent during hydrogen adsorption. As expected, the admittance spectra in Figure 2C show a single semicircle in the double-layer charging region, indicating purely capacitive behavior (see the corresponding equivalent circuit in Figure 2D). However, the admittance spectrum shows two semicircles in the hydrogen adsorption region. The emergence of a new semicircle indicates a second and slow pseudo-capacitive process, hydrogen adsorption/desorption, as represented by the corresponding equivalent circuit in Figure 2D. This second semicircle is not observed for bare Pt(111) in acid media,^29−31^ showing that under those conditions, hydrogen adsorption/desorption is fast and reversible (at least too fast to be measured by EIS). Therefore, we conclude that the presence of graphene on Pt(111) not only changes the energetics of hydrogen adsorption (Figure 1) but also lowers the rate of hydrogen adsorption/desorption. The equivalent circuit (Figure 2D) of double-layer charging can be represented using a pure capacitor (*C*_dl_) in series with the electrolyte resistance, while the adsorption of hydrogen to the Pt electrode can be represented by the addition of an RC parallel circuit, with *R*_ads_ being the adsorption/permeation resistance and *C*_ads_ the adsorption capacitance. *R*_ads_ represents the resistance of proton permeation through graphene, as the electrosorption reaction itself of hydrogen onto the platinum surface is infinitely fast. Therefore, we use variations in *R*_ads_ with experimental conditions as a means to probe the permeation pathway.

To confirm the idea that proton transport through Pt-supported graphene is mediated by defects, the permeation resistance (*R*_ads_) is studied as a function of defect density. The defect density is varied via defect generation and “defect healing” or “defect blocking”. The “defect blocking” is performed by depositing gold in the defect site by cycling between 0.0 and 0.6 V in the 0.1 M H_2_SO_4_ + 5 μM AuHCl_4_.^32^ The advantage of using gold to block the defect is that it has no interfering electrochemical signal in the hydrogen electrosorption region. The defect generation is performed by graphene oxidation–reduction cycles between 0.0 and 1.20 V at 50 mV/s in 0.1 M H_2_SO_4_, as it is well known that oxidation–reduction cycles generate point defects in the graphene surface.^33^

**Figure 3:**
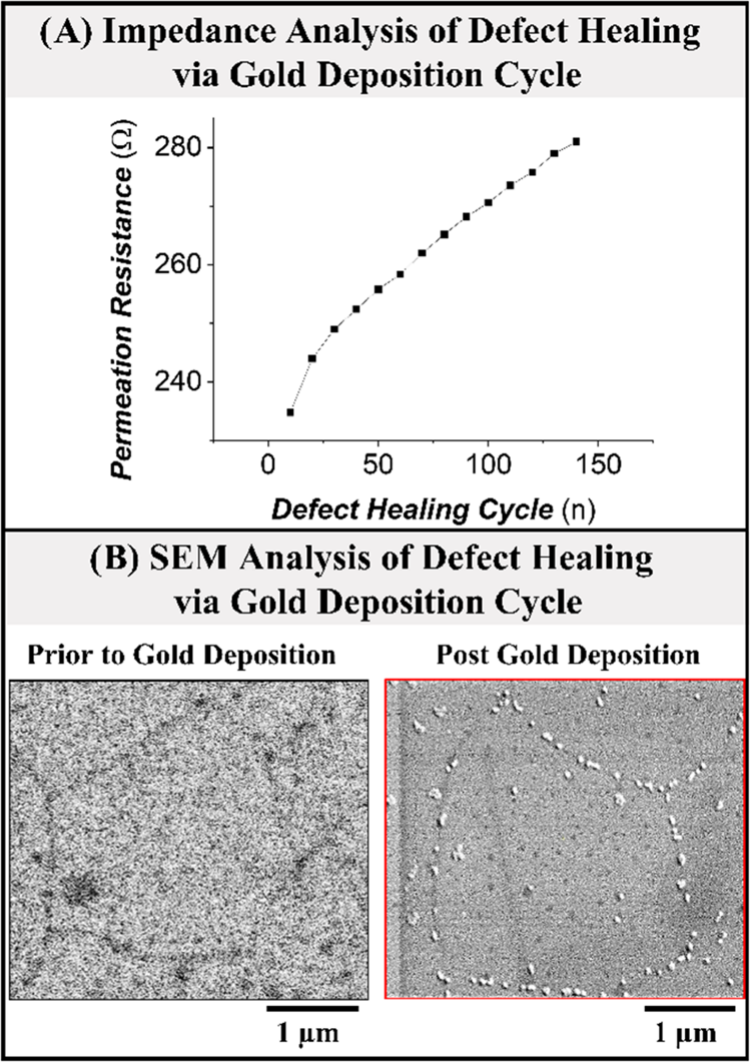
Effect of graphene defect healing on proton permeation resistance. (A) Effective proton permeation resistance at 0.15 V due to defect blocking by cycling between 0.0 and 0.6 V in 0.1 M H_2_SO_4_ + 5 μM AuHCl_4_. (B) SEM images of graphene-covered Pt(111) before and after gold electrodeposition (120 cycles).

Figure 3 shows the effect of defect density on the proton permeation resistance for G-Pt(111) electrode during defect blocking. Figure 3A shows an increase in proton permeation resistance measured at 0.15 V due to defect blocking by gold. Upon potential cycling (0.0–0.60 V, 50 mV/s) of the G-Pt(111) electrode in the gold-containing solution, the amount of gold deposited increases with the number of cycles, as a result of which the apparent defect density decreases.^32^Figure 3B shows two ex situ scanning electron microscopy (SEM) images of G-Pt(111) before and after defect blocking via gold deposition in 120 cycles (Figure 3A). It is observed that gold preferentially electrodeposits on the grain boundaries between the graphene domains, suggesting that these are the active sites for electrochemical reactions on the as-prepared graphene.^32^ Furthermore, the observation that in the presence of gold, the proton permeation of graphene is reduced, indicates that the electrodeposited gold in the domain boundary blocks the access of protons to the Pt(111) surface.

**Figure 4:**
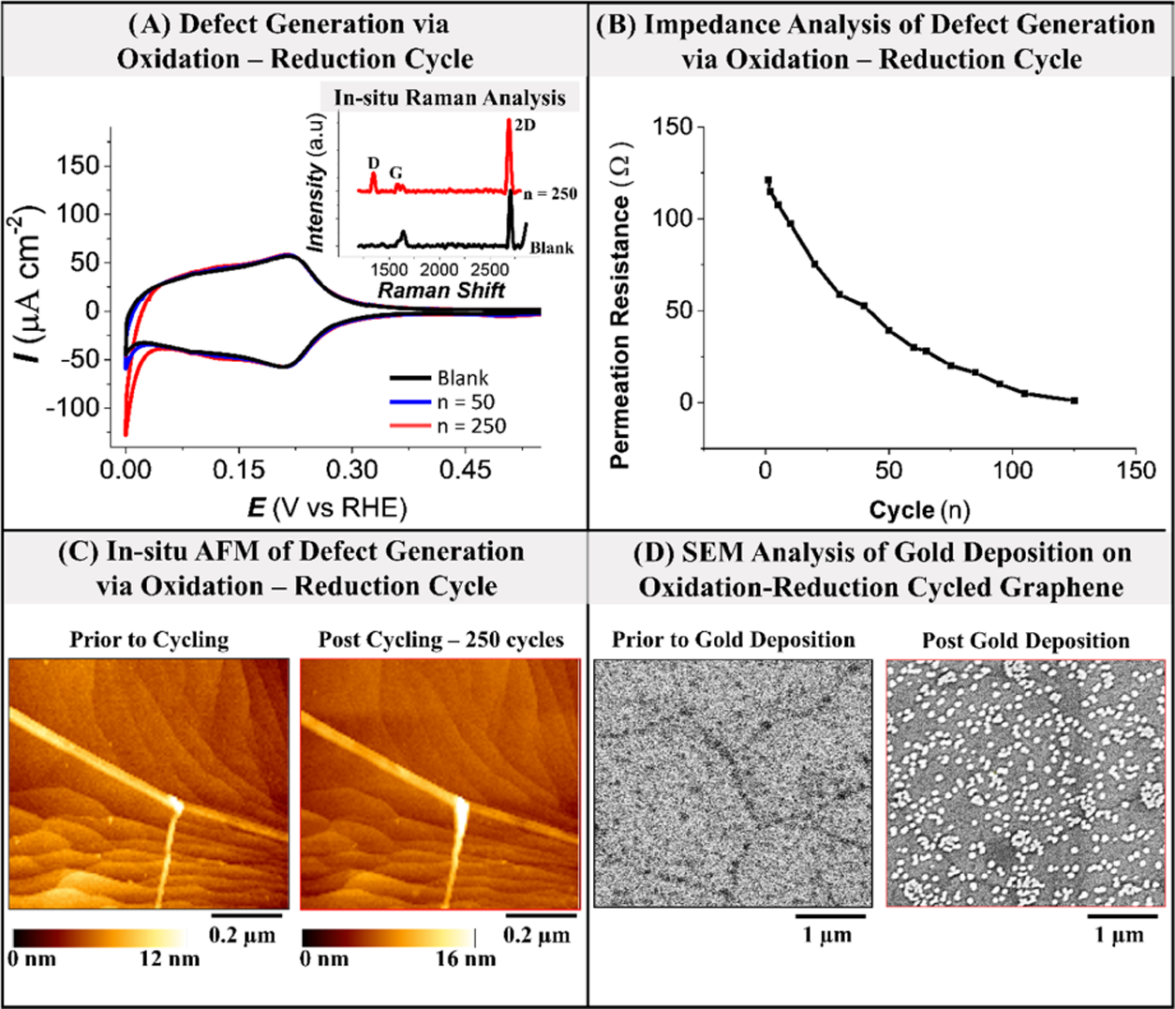
Effect of graphene defect generation on proton permeation resistance. (A) Cyclic voltammogram of G-Pt(111) upon potential cycling (inset—in situ Raman spectra upon cycling). (B) Effective proton permeation resistance at 0.15 V due to defect generation. (C) In situ AFM images before and after potential cycling. (D) SEM images before and after gold deposition on defective graphene-covered Pt(111).

Figure 4 shows the modification of graphene upon defect generation by oxidation–reduction cycling. Figure 4A shows the transformation in cyclic voltammogram on G-Pt(111) upon cycling between 0 and 1.2 V. As the number of cycles (n) increases, the onset of hydrogen evolution moves toward more positive potentials. The inset in Figure 4A shows the in situ Raman spectra of G-Pt(111) upon potential cycling. The Raman spectra show the graphene defect peak D arising due to the potential cycling. In turn, Figure 4B shows that the defect generation decreases the proton permeation resistance measured at 0.15 V. Defect density here is quantified by the number of oxidation–reduction cycles used to generate them, as we cannot quantify the defect density directly. The inset in Figure 4B shows the change in the permeation resistance with the number of cycles (*n*). It is evident that the resistance gradually decreases upon cycling. In addition, we performed in situ atomic force microscopy (AFM) analysis (Figure 4C) of G-Pt(111) to observe the morphological changes during potential cycling. In situ AFM is not sensitive enough to capture atomic defect generation in graphene during potential cycling, but it is able to show that the substrate (Pt(111)) appears essentially unroughened. Therefore, the change in the impedance is attributed to the modification in the graphene layer and not the substrate. We note the overall capacity of G-Pt(111) is not dependent on defect density, as can also be seen from the voltammetry (as the total charge corresponding to H adsorption does not change). We also note that the *C*_dl_ of G-Pt(111) is much lower than that of clean Pt(111), which is also evident from the very low double-layer current of G-Pt(111). Finally, we deposited gold on defect-generated graphene (250 cycles in the potential range of 0–1.2 V), as shown in Figure 4D. In the case of defect generation, the AFM/SEM is not sensitive enough to show the atomic defects in graphene. However, after gold has been deposited electrochemically on such defective graphene, the SEM image (Figure 4D) shows that gold electrodeposition occurs not only along domain boundaries but also inside the domains. This indicates that the oxidation–reduction cycles generate point defects in graphene. Comparing gold deposition on pristine (Figure 3B) and defective graphene (Figure 4D), we conclude that the electrochemical contact mainly occurs via domain boundaries and point defects. From the clear correlation with the permeation resistance, we conclude that these domain boundaries and defects are the pathways for proton permeation.

### Anion Interaction with G-Pt(111)

**Figure 5:**
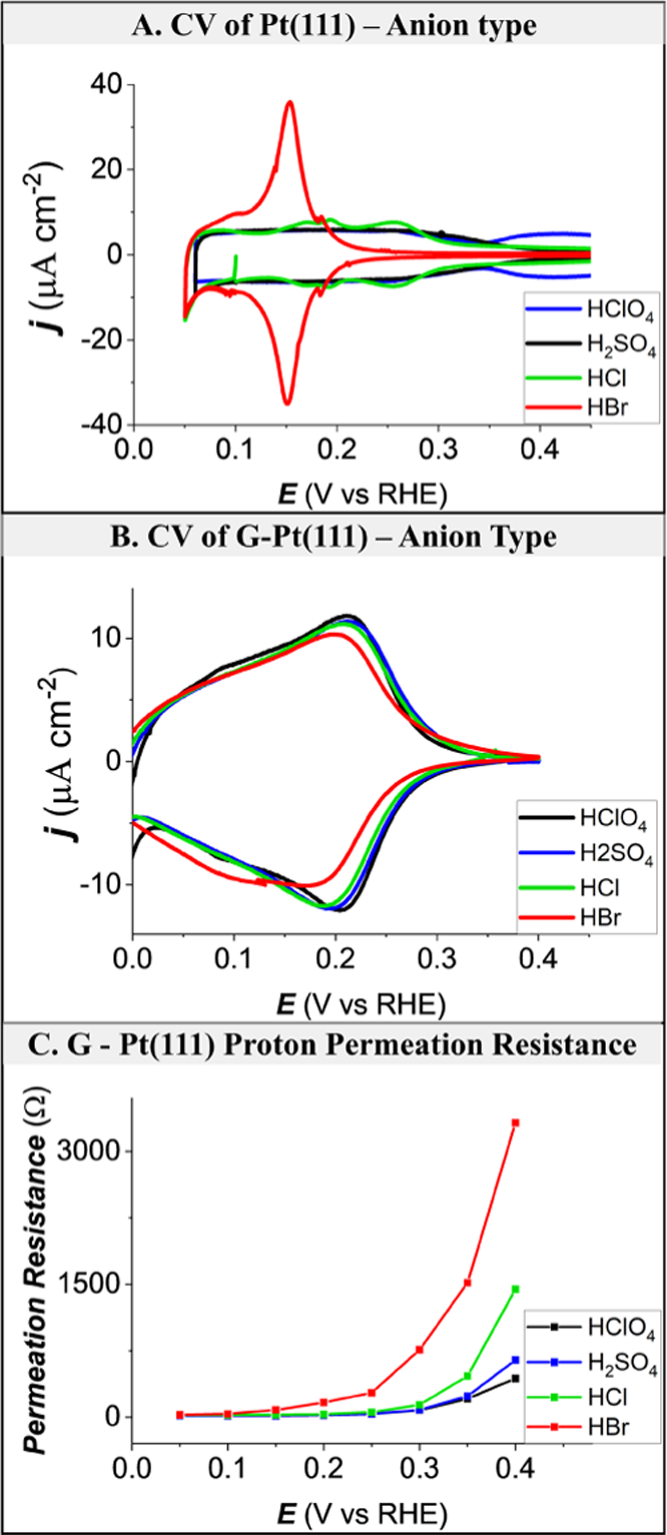
Comparison of SO_4_^2–^, Cl^–^, and Br^–^ adsorption at the Pt(111) and G-Pt(111) electrode from 0.1 M of HClO_4_, H_2_SO_4_, HCl, and HBr solutions, respectively. (A) Cyclic voltammograms of Pt(111) in various electrolytes at a sweep rate of 10 mV/s. (B) Cyclic voltammograms of G-Pt(111) in various electrolytes at a sweep rate of 10 mV/s. (C) Proton permeation resistance for different electrolytes as a function of potential on G-Pt(111) electrode.

In the case of Pt(111), anions often coadsorb specifically along with hydrogen.^34−36^Figure 5 shows the influence of various anions on the hydrogen adsorption at the Pt(111) and G-Pt(111) electrodes. Figure 5A shows the Pt(111) cyclic voltammogram and the anion coadsorption coverage in various electrolytes. In the case of 0.1 M HClO_4_ (no anion coadsorption), the hydrogen adsorption/desorption region appears between 0.06 and 0.4 V (vs RHE). In the presence of anions (SO_4_^2–^, Cl^–^, Br^–^), the voltammetric profiles are significantly different because of the specific coadsorption of anions. The potential at which the adsorption of each anion starts increases in the order: bromide (∼0.1 V vs RHE),^34,37,38^ chloride (∼0.25 V vs RHE),^39,40^ and (bi)sulfate (∼0.35 V vs RHE).^41,42^Figure 5B,C shows the G-Pt(111) cyclic voltammogram and the proton permeation resistance across graphene in various electrolytes. The CVs in all electrolytes show only hydrogen adsorption (no anion coadsorption) since the graphene cover blocks all species other than protons (anions, cations, and water molecules). On the other hand, Figure 5C shows that anions (SO_4_^2–^, Cl^–^, Br^–^) influence the proton permeation resistance. This is indirect evidence that the anion still adsorbs near the graphene defect site and blocks the proton permeation. Interestingly, similar to Pt(111), the potential region in which the adsorption of anions influences the proton permeation increases in the order: bromide (∼0.1 V vs RHE), chloride (∼0.25 V vs RHE), and (bi)sulfate (∼0.35 V vs RHE).

**Figure 6:**
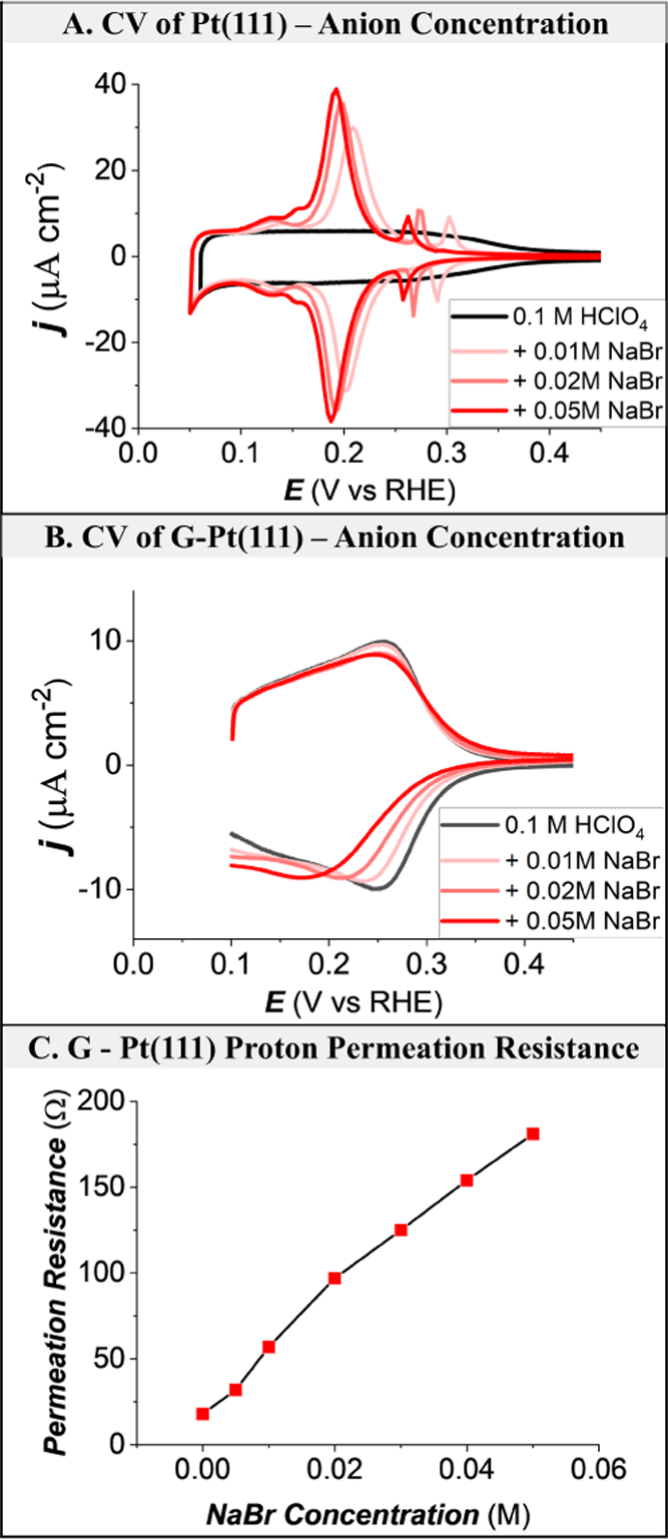
Comparison of Br^–^ adsorption at the Pt(111) and G-Pt(111) electrode from various concentrations. (A) Cyclic voltammograms of Pt(111) in various 0.1 M HClO_4_ + *x* M NaBr electrolytes at a sweep rate of 10 mV/s. (B) CVs of G-Pt(111) measured in 0.1 M HClO_4_ + *x* M NaBr at the rate of 10 mV/s. (C) Proton permeation resistance of G-Pt(111) measured at 0.2 V in 0.1 M HClO_4_ + *x* M NaBr. Note: the kinetics of the bromide adsorption/desorption in panel (B) is different from that in Figure 5B, presumably due to a different G-Pt(111) sample and a different electrolyte.

Figure 6 represents the cyclic voltammetry curves (CVs) and proton permeation resistance of a G-Pt(111) electrode in 0.1 M HClO_4_ solutions with different concentrations of added NaBr. Figure 6A shows the CVs of Pt(111), and Figure 6B shows the CVs of G-Pt(111). For G-Pt(111), upon NaBr addition, there is no new peak, but the hydrogen adsorption peak becomes less reversible due to sluggish adsorption kinetics. The slow kinetics can be attributed to blockage by bromide ions of defect sites in graphene. In the case of bare Pt(111) (Figure 6A), the bromide coadsorption peak is apparent. To corroborate, impedance spectra are measured at 0.2 V, and it is evident from Figure 6C that the proton permeation resistance increases with successive additions of NaBr.

### Density Functional Theory Calculations

**Figure 7:**
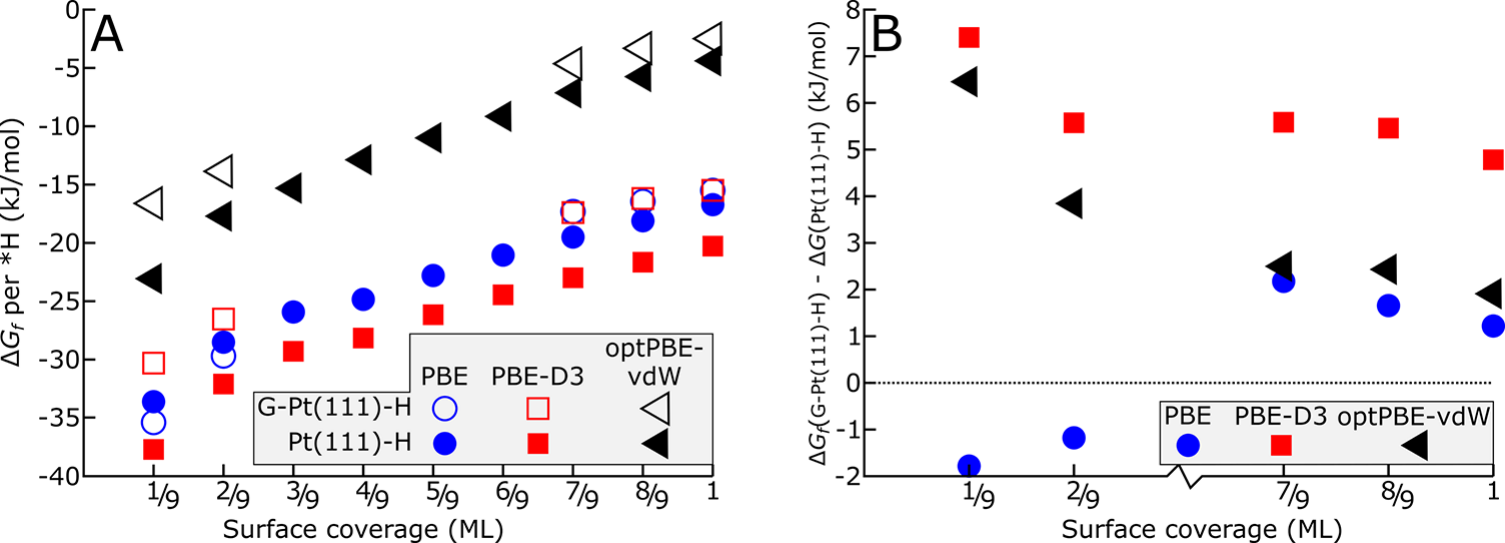
Hydrogen binding energies as a function of *H-coverage. (A) Comparison of *H binding energies on pristine Pt(111) and G-Pt(111) for different functionals. All functionals show a monotonic decrease of H* binding energy for increasing coverage, both for pristine Pt(111) and graphene-covered Pt(111), and show the *H binding energy difference between G-Pt(111) and Pt(111) generally decreasing with increasing coverage. (B) Graphene overlayer effect on the binding energies for different functionals. PBE shows no net graphene effect, while optPBE-vdW and PBE-D3 show significant *H binding energy weakening at low *H coverages and all *H coverages, respectively.

To support the experimental findings with computational evidence, DFT energies were computed for Pt(111) with and without a graphene overlayer and with various *H coverages, as detailed in the Computational Methods section. To measure the effect of van der Waals (vdW) interactions and check whether the graphene overlayer-related shifts in *H binding energy are consistent across exchange–correlation functionals, three functionals were used for the relaxations of each system: the PBE functional,^43^ the PBE functional with DFT D3 dispersion corrections (PBE-D3) by Grimme et al.,^43,44^ and the optPBE-vdW functional.^45^ All energies are listed in Tables S1 and S2. The corresponding binding energies for *H as a function of coverage are visualized in Figure 7A. The binding energy differences between *H with and without graphene are shown in Figure 7B.

In Figure 7A, we observe that all functionals show a gradual and monotonic decrease of *H binding energy for increasing coverages, both for pristine Pt(111) and G-Pt(111). PBE shows no net effect of graphene on the *H binding energy (0 ± 3 kJ/mol). This is consistent with the absence of interactions between graphene and Pt(111) for PBE, as shown in Table S2. For PBE-D3, *H binding is uniformly weakened across different coverages by 6 ± 2 kJ/mol, while for optPBE-vdW, there is a weakening of 7 kJ/mol at lower *H coverages, which trails off to ∼2 kJ/mol for *H coverages near 1 ML. The latter is within the error margin of PBE. For both PBE-D3 and optPBE-vdW, we observe a H–Pt bond weakening effect due to the presence of graphene, in line with the experimentally observed weakening in Figure 1. The fact that this weakening is not observed for PBE strongly suggests that it is primarily due to the decrease of the van der Waals interaction of the graphene layer with Pt(111). The weakening with respect to H on Pt(111) and pristine G-Pt(111) is partly caused by the increased distance between the graphene layer and conducting Pt surface, which is itself effectuated by repulsion by the H atoms. The effect vdW interactions have on *H binding under graphene is discussed in the Supporting Information, on the basis of a thorough comparison of PBE and PBE-D3. Besides, for all functionals, the coverage dependence is steeper for the Pt(111) than for the G-Pt(111), showing the lateral interactions between adsorbed hydrogen atoms are less repulsive in the presence of a graphene overlayer. This is also in good agreement with experimental observations in Figure 1B. Yet, optPBE-vdW appears to underbind *H consistently, since the total free energy of adsorbed H becomes more positive when adding H beyond 5/9 ML. The potentials for 1/9 ML *H formation on graphene-covered Pt(111) predicted by PBE and PBE-D3 are 0.35 and 0.39 V (vs RHE), similar to the observed *H onset at 0.40 *V*_RHE_, while the predicted onset potential using optPBE-vdW is 0.24 *V*_RHE_. Since graphene favorably binds to Pt(111) for PBE-D3 and optPBE-vdW, whereas the latter functional deviates from experimental observations regarding *H adsorption, the PBE-D3 functional is used in all subsequent calculations.

Subsequently, barriers were calculated for the diffusion of *H on pristine Pt(111) and G-Pt(111). For this purpose, we assumed that *H jumps between fcc and hcp sites across bridge sites on both pristine Pt(111) and graphene-covered Pt(111), the energies of which are listed in Table S3. Calculated diffusion barriers amount to 5 kJ/mol for *H on pristine Pt(111) and to 9 kJ/mol for *H at the interface between graphene and Pt(111). These barriers are not significant and suggest that once adsorbed, *H can easily cover the entire Pt(111) surface, even when covered by a graphene layer, in agreement with experimental observations.

Previous calculations performed by Tsetseris et al. and Mazzuca and Haut showed that atomic hydrogen cannot move classically through defect-free graphene,^46,47^ with the latter arguing that tunneling is necessary for hydrogen transport.^47^ However, neither study fully describes H diffusion through Pt(111)-bound graphene,^46,47^ and therefore, we verified these calculations for G-Pt(111). The energies of a hydrogen atom moving from vacuum through intact graphene onto Pt(111) are shown in Figure S4. The calculated barrier for this process exceeds 190 kJ/mol, which means the process is unlikely to occur under these conditions. Hydrogen permeation through graphene hydrogenation, in which hydrogen reacts to graphene first and is subsequently transported into the space between graphene and Pt(111), is not likely either, with a formation energy of no less than 123 kJ/mol for graphene functionalization on the vacuum interface. This corresponds to the near-absence of proton transport for defect-free graphene, i.e., for low numbers of oxidation–reduction cycles, as shown in Figure 4B. Young et al. observed that D_2_ diffusion through graphene is likely accommodated by atomic-scale defects.^48^ For atomic hydrogen diffusion, however, such as the reduction of solvated H^+^ from the solvent, the interaction of atomic-scale graphene defects with the Pt(111) surface may affect the effective permeability of, and transport from, these vacancies. The smallest atomic-scale defects in graphene include monovacancies and divacancies.

**Figure 8:**
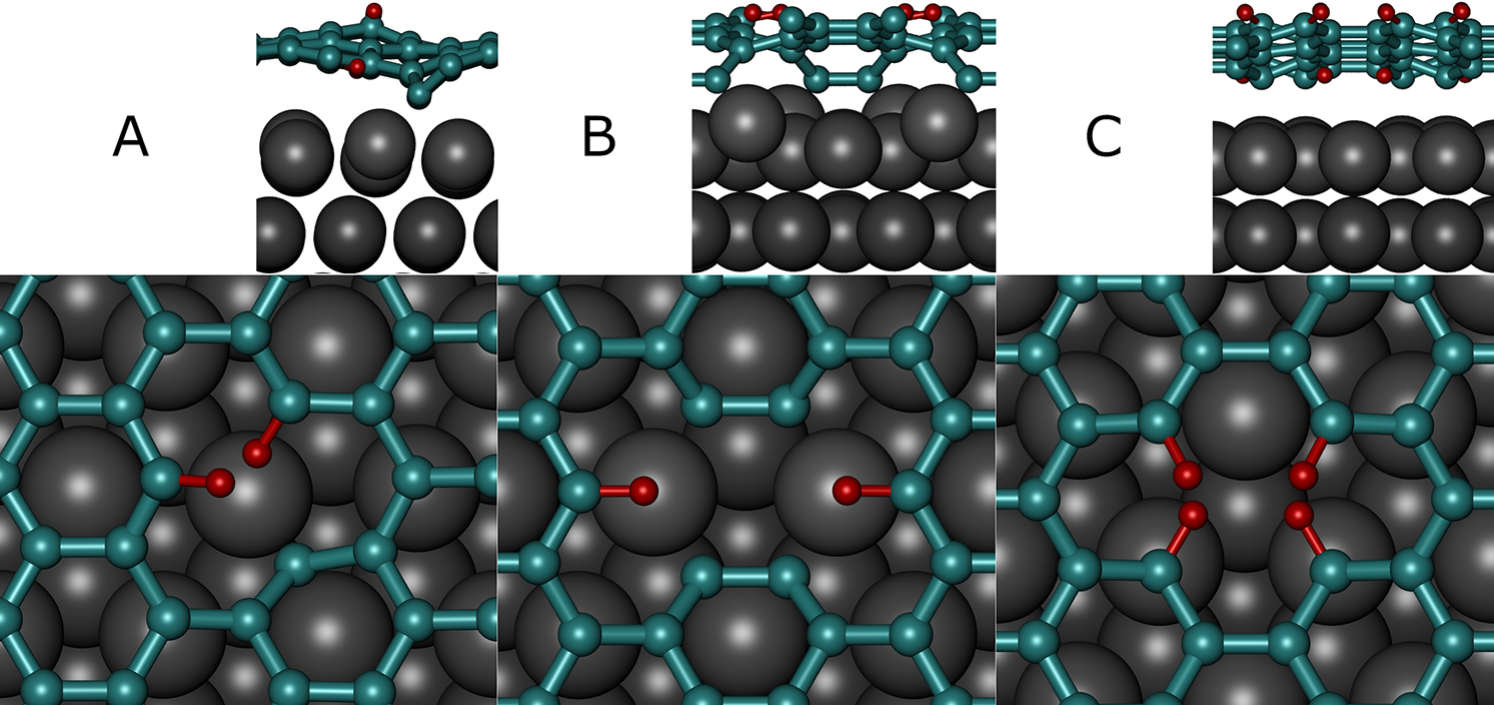
Thermodynamically most stable hydrogen-passivated vacancies in lateral (above) and top (below) views. Pt, C, and H are shown in gray, cyan, and red, respectively. (A) Monovacancy. (B) Far divacancy. (C) Close divacancy.

Following the idea that graphene vacancies are necessary for hydrogen atom permeation from one side of the graphene layer to the Pt(111) surface, we computed the energies of formation for different hydrogen-passivated monovacancies and divacancies, as listed in Table S5 and described in the Computational Methods section. Based on these data, at least 2 out of 3 terminal C atoms of the monovacancy are hydrogenated under standard conditions (see Figure 8A), while 2 out of 6 terminal C atoms of the “far divacancy” (see Figures 8B) and 4 out of 4 terminal C atoms of the “close divacancy” (see Figure 8C) are hydrogenated.

**Figure 9:**
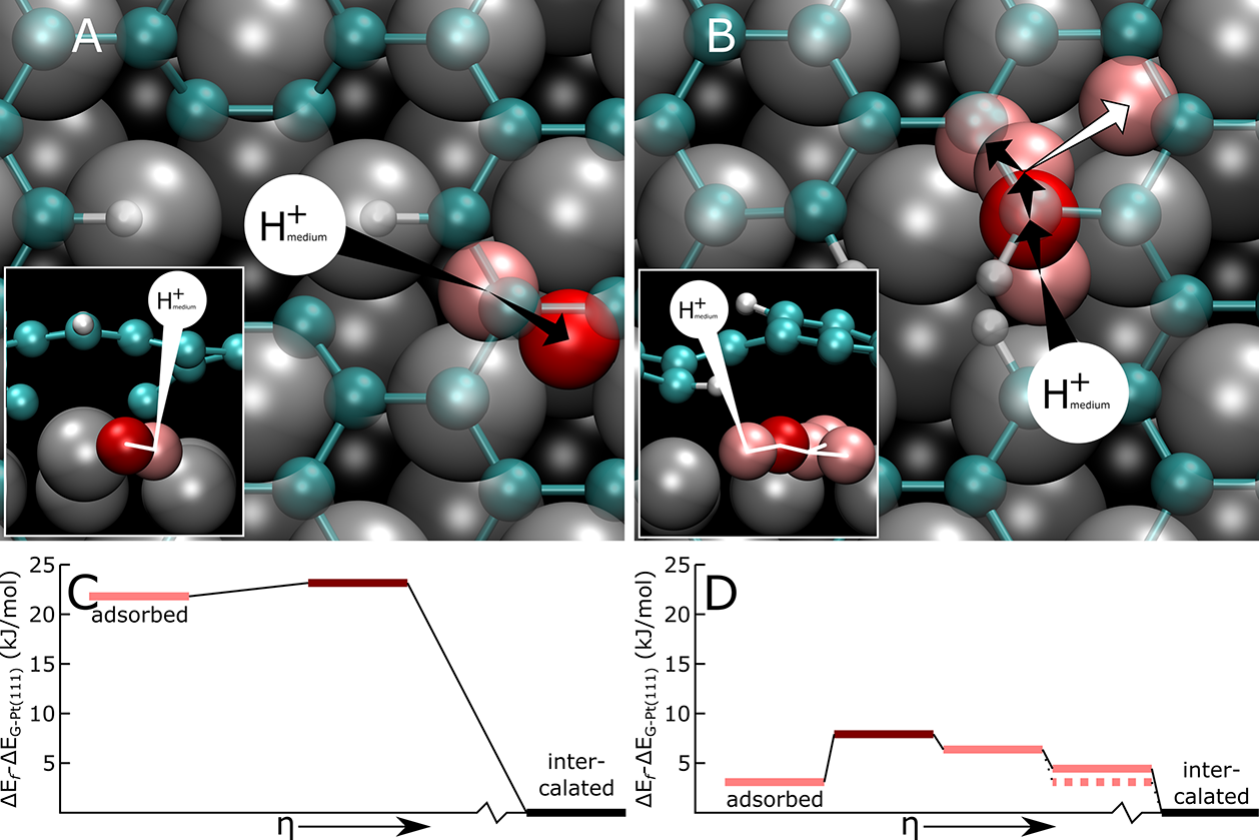
Lowest-barrier permeation pathways through graphene vacancies for *H vs generalized reaction coordinate η. (A, B) Top-view illustrations of *H transport through far and close divacancies, respectively, with side view parallel to carbon vacancies in insets. Carbon atoms are shown in cyan; graphene vacancy-bound hydrogen atoms are white. Surface platinum atoms are shown in light gray, dark gray, and black for the top, hcp, and fcc sites, respectively. H^+^ in the medium is referred to using a large white circle, while *H is shown in red at the transport barrier and in pink elsewhere. The expected direction of transport is shown using arrows. (C, D) Thermodynamic description of lowest-barrier *H permeation pathways through far and close divacancies, respectively. Reaction coordinate η corresponds to the order in transport steps displayed in arrows in panels (A) and (B), respectively, and goes from the initial “adsorbed” *H configuration to the (nondepicted) fully “intercalated” *H on G-Pt(111) far from the vacancy. Dark red lines represent the transport barrier, while pink lines correspond to other configurations.

The most stable passivated monovacancy, with a passivation onset potential of 0.26 *V*_RHE_, interacts strongly with Pt(111), while its two C–H bonds occupy the gap left by the vacancy. Hydrogen adsorption transport through, or hydrogen adsorption underneath this gap, is therefore unlikely. Both divacancies at least partially expose the underlying Pt(111) surface to the medium; hence, all adsorption sites for *H underneath these vacancies are taken into account. Their binding energies are listed in Table S4, from which onset potentials can be derived for hydrogenation up to the specific number of passivating hydrogen atoms of a far and close divacancy at 0.83 and 0.99 V, respectively. All onset potentials are in the range of the oxidation–reduction cycles described in the Mechanism of Proton Permeation section above, and passivation is, therefore, likely to occur during vacancy formation in aqueous media. From these binding energies, lowest-barrier permeation pathways were constructed starting on sites exposed to the medium, which are described in Figure 9.

The minimum thermodynamic barrier for *H transport through a far graphene divacancy is 23 kJ/mol, while the equivalent value for a close divacancy is 8 kJ/mol. The weak binding energies of *H under the far graphene divacancy create an effective barrier for *H transport, more than doubling the expected Volmer reaction barrier on an unmodified Pt(111). On the other hand, the binding energies under the upward-pointing C–H bonds allow for permeation of *H, with a barrier close to the diffusion barrier on Pt(111). This low-barrier permeation process through divacancies, combined with the observations on molecular hydrogen permeation through atomic-level vacancies by Young et al.,^48^ supports the hypothesis that hydrogen (atomic and molecular) permeates through point defects and corresponds to the experimental observation that a graphene layer with a great number of point defects will allow for significant permeation and reaction rates.

## Conclusions

In this paper, we have studied the energetics and kinetics of hydrogen electrosorption on Pt(111) covered by a monolayer of graphene. Both experiment and theory show that hydrogen binds more weakly to Pt(111) in the presence of graphene. Density functional theory calculations with various functionals show that this effect is due to Van der Waals interactions; presumably, the presence of hydrogen weakens the binding of the graphene to Pt(111). In addition, the lateral interactions between adsorbed H on Pt(111) become less repulsive in the presence of graphene, as confirmed by both experiments and DFT calculations. Experiments with isolated defects generated by oxidation–reduction cycling and domain boundaries covered by gold clusters show that domain boundaries and defects are the sites where hydrogen permeates the graphene layer. The rate constant for hydrogen adsorption follows directly the defect density, as confirmed by impedance spectroscopy measurements. Although graphene blocks the interaction of anions with the Pt(111) surfaces, anions do adsorb near the defects: the rate constant for hydrogen permeation is sensitively dependent on anion identity and concentration, with the more strongly adsorbing anion (bromide) having the largest effect. DFT calculations confirm that intact graphene has a prohibitively high barrier for hydrogen permeation, that defects allow for a significantly lower barrier, and hydrogen can diffuse on the Pt(111) surface with low barriers, even in the presence of graphene. The improved understanding of the effect of graphene overlayer of Pt, as obtained in this paper, will hopefully improve the design of tailor-made graphene-modified electrodes for specific electrochemical applications.

## Materials and Methods

### Electrodes, Electrolytes, and Electrochemical Cells

The experimental work was conducted using monocrystalline Pt(111) disc working electrodes (WE), and the graphene overlayer on Pt(111) was prepared using the chemical vapor deposition technique.^17^ The electrochemical experiments were conducted in a Pyrex, two-compartment electrochemical cell. A platinum mesh was used as a counter electrode (CE) (99.998% in purity, Aesar), and a reversible hydrogen electrode (RHE) was used as a reference electrode (RE). The glassware was precleaned according to a well-established procedure.^49−51^ The electrolyte solution was prepared from high-purity chemical (Merck-ultrapur grade) and ultrahigh-purity (UHP) water (Milli-Q, Millipore; resistivity ≥ 18.2 MΩ cm). All electrochemical experiments were conducted using a Bio-Logic SP-300 potentiostat using proprietary software. Impedance spectra were measured with frequencies ranging from 10 kHz to 0.1 Hz and a peak-to-peak amplitude of 5 mV. The data were fitted to the equivalent electric circuit (EEC).^29−31^

### In Situ AFM and Raman Analysis

The in situ AFM and Raman experiments were carried out in an electrochemical cell made of PEEK.^52,53^ Before assembly, the cell components were cleaned by sonication in high-purity ethanol and Millipore Milli-Q water (resistivity 18.2 MΩ cm), respectively, and then blow-dried in Ar (g). Before each experiment, the counter electrode Pt foil (99.9%, MaTeck) was flame-annealed and quenched with Milli-Q water before assembling into the in situ cell. An Ag/AgCl (3 M KCl) electrode (WPI) was used as the reference electrode, and the counter electrode was a Pt wire. A potentiostat (μAutolab type III) was coupled with the AFM/Raman spectroscopy to control the electrochemical conditions during the experiments.

The details of in situ AFM instrumentation and operation have been explained elsewhere.^52,53^ The AFM (JPK Instruments) scan rate was 1 Hz, and all of the images were obtained using the tapping mode to minimize the damage to the electrode. The tips used were purchased from Bruker (SNL, resonance frequency: 65 kHz, spring constant: 0.35 N/m). Images were taken either at 0.5 V after potential cycling or during cycling simultaneously with cyclic voltammetry (CV). The electrolyte was prepared from H_2_SO_4_ (Merk Ultrapur, 96%), which was neither thoroughly degassed nor refreshed during the experiment. The immersion Raman spectra were collected using a WITEC α 300 R-Confocal Raman Imaging using immersion with a laser wavelength of 532 nm. The electrochemical cell design for immersion Raman spectroscopy is based on the laboratory setup described elsewhere.^54,55^ To minimize the potential damage from the laser heating effect, the laser power was controlled under 1.1 mW. All measurements were performed under ambient conditions at room temperature.

### Ex Situ Analysis

Graphene morphology was analyzed using scanning electron microscopy (SEM) (Apreo, Thermo Scientific) and atomic force microscopy (AFM) (JPK Nanowizard 4). The SEM is operated in high vacuum condition (<1 × 10^–6^ mbar), and the images are collected at a beam setting of 10 kV and 0.40 nA using an Everhart–Thornley detector.

## Computational Methods

The DFT calculations were carried out in VASP 5.4.4^56^ and using the projector augmented-wave method.^57^ The fcc Pt(111) surface slabs were modeled using four layers of (3 × 3) Pt atoms. For calculating the net binding energy on pristine Pt(111), various (0–9 atom) coverages of *H in all possible symmetry-independent on-surface adsorption configurations were added onto the top layer. Similarly, various 0, 1, 2, 7, 8, and 9 *H atom coverages were placed on top of the Pt(111) slab and covered by a commensurate 2√3 × 2√3 *R*30° graphene overlayer, as described in Section S.1 in the Supporting Information. All systems were relaxed to a maximum atomic force of 0.02 eV/Å, with a 450 eV plane-wave cutoff and a 6 × 6 × 1 γ-centered Monkhorst-Pack *k*-point grid.^59^ The free energies of adsorption of *H were calculated with the computational hydrogen electrode model using the individual DFT energies and configurational entropies of each individual configuration^58^ and vibrational corrections, as described in the Supporting Information (Section S.1.2). Effect of solvation was not included in the calculations.

Barriers for *H diffusion were assessed by calculating the free energy profile between the lowest and second-lowest energy adsorption sites. We also computed the energetics of proton permeation underneath passivated graphene defects. First, either one carbon atom is removed from the graphene layer forming a monovacancy, or two atoms along the long or short edges of a graphene hexagon. Subsequently, the now-available carbon atoms adjacent to the vacancy are passivated with hydrogen atoms in various stoichiometries and configurations and relaxed. Finally, we added single hydrogen atoms in all unique adsorption sites under the vacancies and obtained the resulting energies, through which the permeation pathways and energetics can be determined.

## Supporting Information Available

Thermodynamic data obtained using the methods are listed in the density functional theory calculations subsection of the experimental and computational methods section of the main text, and the computational analysis of the data and details of the computations and computational procedures are described (PDF)
