# Supplementary material for: Energetics and Kinetics of Hydrogen Electrosorption on a Graphene-Covered Pt(111) Electrode
Source: JACS Au. 2023 Jan 18;3(2):526–35. doi: 10.1021/jacsau.2c00648 (PMC9976337; doi:10.1021/jacsau.2c00648)
Supplement: Supplementary file 1 — au2c00648_si_001.pdf [file au2c00648_si_001.pdf]

Supplementary Material to

Energetics and kinetics of hydrogen electrosorption on a graphene-covered Pt(111) electrode

Nakkiran Arulmozhi[1], Selwyn Hanselman[1] , Viorica Tudor[1], Xiaoting Chen[1], David van Velden[1], Grégory F. Schneider[1], Federico Calle-Vallejo[2], Marc T. M. Koper[1]*

[1] Leiden Institute of Chemistry, Leiden University, PO Box 9502, Leiden 2300 RA, The Netherlands

[2] Department of Materials Science and Chemical Physics & Institute of Theoretical and Computational Chemistry (IQTCUB), University of Barcelona, Martí i Franquès 1, 08028 Barcelona, Spain.

In this Supplementary Material, we list the thermodynamic data obtained using the methods listed in the Density Functional Theory Calculations subsection of the Experimental and Computational Section of the main text, and describe the computational analysis of the data. All energies are provided in eV.

S.1. Hydrogen and graphene configurations on Pt(111)

*S.1.1. *H configurations on Pt(111)*

All 1 *H configurations on the periodic 3×3 Pt(111) slab are generated by placing a hydrogen atom on all locally symmetric surface sites (on-top, bridge, hcp, fcc), as illustrated in Figure S1A. Similarly, all unique 3-fold symmetric graphene configurations on the Pt(111) slab are generated by centering one of the carbon atoms of the graphene overlayer on the 3-fold symmetric surface sites: on-top, hcp, or fcc. These are shown in Figure S1B-D, respectively. Note that a bridge-centered graphene overlayer would be identical to the on-top-centered graphene overlayer and is, therefore, excluded.

The two lowest energy 1 H binding sites for each functional are subsequently used to generate all possible configurations of 2-8 *H on the Pt(111) slab. Additionally, 9 *H all-top, all-hcp, and all-fcc configurations are used to verify whether the two most stable binding sites for 1 *H are also most stable near a 1 ML coverage limit. All configurations which have two *H atoms directly adjacent to one another, in the closest top-bridge, top-hcp, top-fcc, bridge-fcc, bridge-hcp, and hcp-fcc site pairs, are excluded. Of all symmetry-equivalent configurations, only one is calculated to reduce the total computational expense. Symmetry-equivalent, in this context, means that two adsorption configurations can be transformed into one another by applying any product of symmetry operations which would transform the Pt(111) slab into itself. These symmetry operations are mirroring with respect to the plane perpendicular to the slab intersecting its farthest corners, 3-fold rotation with respect to the origin, and translation by one Pt-Pt atom distance along either surface lattice vector. The total number of unique yet symmetry-equivalent configurations within each set is used to calculate the configurational entropy, $S_{\text{conf}} = k_B \ln(W_{\text{conf}})$.

Fig. S1.

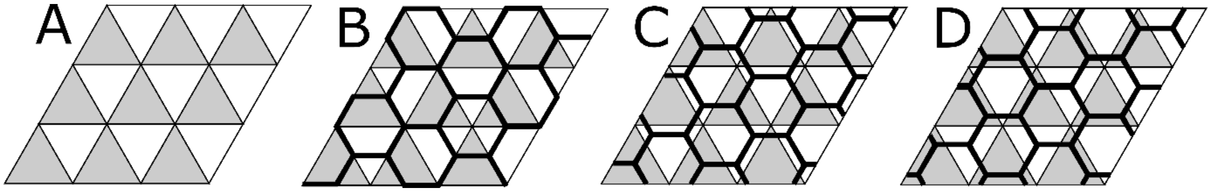

Hydrogen and graphene binding sites on Pt(111). A) 3×3 Pt(111) surface. Line intersections represent on-top sites, lines represent bridge sites, grey and white triangles represent hcp and fcc sites, respectively. B) Pt(111) with a top-centered graphene overlayer. C) Pt(111) with an hcp-centered graphene overlayer. D) Pt(111) with an fcc-centered graphene overlayer.

S.1.2. Hydrogen and graphene configurations for graphene-covered Pt(111)

Similarly, 1 and 9 *H configurations are calculated on individual sites (top, bridge, hcp, and fcc) on the graphene-covered Pt(111) surface. When a graphene carbon atom is centered above a specific site, there are two unique types of adsorption site corresponding to the graphene site as shown in Figure S1: one under a graphene carbon atom, and another under a graphene ring. From the most stable 1 *H site, 2 *H configurations are generated, while for the 7 *H and 8 *H configurations, two or one H atom(s) are removed from all stable 9 *H configurations, respectively.

S.2. Thermodynamics

S.2.1. Definition of thermodynamic methods

Energies of formation for $H_2(g)$ and all adsorbed *H configurations on the pristine or graphene-covered Pt(111) surface were calculated, with or without vibrational free-energy corrections, with respect to the ground state at 300 K and standard pressure, using computational parameters described in the main text. For *H on pristine Pt(111), formation energies (excluding *H vibrational free energy corrections) are defined with respect to $H_2(g)$ and the pristine surface, as in the computational hydrogen electrode model by Nørskov *et al.* (*1*):

$$n* + \frac{n}{2}\mathrm{H}_2 \rightarrow n^*\mathrm{H} \qquad (S1)$$

as

$$\Delta E_{f,n,i} = U\left(\mathrm{PtH}_{n,i}\right) - U(\mathrm{Pt}) - \frac{n}{2}G\left(\mathrm{H}_2(g)\right) \qquad (S2)$$

where $U(x)$ is the DFT energy of the ground state of species x, $\mathrm{PtH}_{n,i}$ is the slab with n *H in configuration i, and Pt is the (3×3) Pt(111) slab. This formation energy does not yet include vibrational free-energy corrections for *H. These corrections for *H were calculated using single-atom displacement methods (0.02 Å) under the harmonic approximation on all *H atoms for specific 1, 2, 7, 8, and 9 *H configurations on the (3×3) Pt(111) slab. For 1 *H, the two configurations with lowest E_f per functional were analyzed, yielding their $G_{vib,1,i}$. For 2, 7, 8, and

9 *H, we selected the configurations to be analyzed by calculating the relative probability of finding such configuration for its specific coverage using Boltzmann statistics:

$$P_{f,n,i} = \frac{z_{f,n,i}}{Z_{f,n}} = \frac{W_{f,n,i} \exp\left(\frac{-\Delta E_{f,n,i}}{k_B T}\right)}{\sum_j W_{f,n,j} \exp\left(-\frac{\Delta E_{f,n,j}}{k_B T}\right)} = \frac{\exp\left(\frac{-\Delta E_{f,n,i} + TS_{conf,n,i}}{k_B T}\right)}{\sum_j \exp\left(-\frac{\Delta E_{f,n,j} + TS_{conf,n,j}}{k_B T}\right)} \quad \text{(S3)}$$

where $W_{f,n,i}$ is the number of configurations which are symmetry-equivalent to configuration n, i and $S_{conf,n,i}$ is the corresponding configurational entropy. For each coverage, we chose the most probable configurations together representing a total probability greater than 50% for that specific coverage and functional. Additional vibrational calculations are performed if the 1 *H calculations for a functional suggest that one specific site is energetically less stable yet vibrationally favored, and the positive internal energy difference at greater coverages may be cancelled out by the vibrational free energy. After calculating the vibrational free energies $G_{vib,n,i}$ for each mode for a specific coverage, we obtained the weighted $G_{vib,n}$ by multiplying the individual $G_{vib,n,i}$ with their adjusted vibrational contributions $P_{vib,n,i}$:

$$G_{vib,n} = \sum_i P_{vib,n,i} G_{vib,n,i} = \sum_i \frac{z_{vib,n,i}}{Z_{vib,n}} G_{vib,n,i} = \sum_i \frac{\exp\left(\frac{-\Delta E_{f,n,i} + TS_{conf,n,i} - G_{vib,n,i}}{k_B T}\right)}{\sum_j \exp\left(-\frac{\Delta E_{f,n,j} + TS_{conf,n,j} - G_{vib,n,j}}{k_B T}\right)} G_{vib,n,i} \quad \text{(S4)}$$

The vibrational free energies per *H near these limits for PBE, PBE-D3, and optPBE-vdW amount to 0.15 ± 0.02 eV, 0.15 ± 0.01 eV, and 0.14 ± 0.00 eV, respectively, all based on predominantly fcc-bound configurations. Since the maximum errors in the vibrational free energies are similar to or smaller than 0.01 eV and there is no coverage dependence, the mean vibrational free energies per *H are used for all coverages as vibrational corrections per *H: $G_{vib,corr}$. For optPBE-vdW, E_f for top-bound *H is lower than for fcc-bound *H, while $G_{vib,i,n}$ for top-bound configurations is 0.18 ± 0.02 eV instead. Hence, for configurations calculated with optPBE-vdW for which the vibrational energy is not directly calculated, an additional correction $\Delta E_{f,top}$ of 0.04 eV is added to each top-bound *H. For other functionals, $\Delta E_{f,top}$ is 0.00 eV. Weighted energies $E_{f,n}$, which are free energies based on internal energies and configurational entropies, are calculated using the following formula:

$$\Delta E_{f,n} = -k_B T \ln\left(\sum_i z_{fcorr,n,i}\right) = -k_B T \ln\left(\sum_i exp\left(-\frac{\Delta E_{f,n,i} - TS_{conf,n,i} + n_{top}\Delta E_{f,top}}{k_B T}\right)\right) \quad \text{(S5)}$$

where n_{top} is the number of top-bound *H. From these weighted internal energies, the overall free energy for *H coverage n is calculated:

$$\Delta G_{Pt,n} = \Delta E_{f,n} + nG_{vib,corr}. \quad \text{(S6)}$$

Similarly, we calculated the free energy of *H on graphene-covered (3×3) Pt(111) for 1, 2, 7, 8, and 9 *H coverages. For graphene and *H on graphene-covered Pt(111), formation energies (excluding *H vibrational free energy corrections) are defined with respect to $H_2(g)$, graphene in vacuum, and the pristine surface:

$$n * + \frac{n}{2}\mathrm{H_2} + \mathrm{gr} \rightarrow \mathrm{gr^*H}_n \quad \text{(S7)}$$

as

$$\Delta E_{f,n,i,j} = U(\mathrm{grPtH}_{n,i,j}) - U(\mathrm{Pt}) - U(\mathrm{gr}) - \frac{n}{2} G(\mathrm{H_2}(g)) \quad \text{(S8)}$$

where $U(x)$ is the internal (or DFT) energy of the ground state of species x, $grPtH_{n,i}$ is the slab with n *H in configuration i and graphene adsorbed on site j, Pt is the (3×3) Pt(111) slab, and gr is the graphene overlayer. This time, the graphene orientation yielding the lowest $\Delta E_{f,n,i,j}$ for each coverage and configuration was selected for vibrational analysis. The vibrational mode energies of the adsorbed hydrogen atoms were calculated using 0.02 Å single-atom displacements under the harmonic approximation. Contrary to Pt(111), however, G_{vib} values are calculated for all *H configurations within each coverage. Again, we calculated all *H and graphene adsorption configurations described in Section S.1, and selected the most stable graphene adsorption site for each hydrogen adsorption site (i.e. $\Delta E_{f,n,i}$) for calculating their vibrational free energies, $G_{vib,n,i}$. This time, the total free energy of each of these *H configurations is used to calculate a cumulative free energy for its corresponding *H coverage:

$$\Delta G_{f,n} = -k_B T \ln\left(\sum_i z_{f,n,i}\right) = -k_B T \ln\left(\sum_i exp\left(-\frac{\Delta E_{f,n,i} - TS_{conf,n,i} + G_{vib,n,i}}{k_B T}\right)\right) \quad \text{(S9)}$$

where $S_{conf,n,i}$ is the configurational entropy of the *H configuration. From these values, first the formation energy of the *H configuration onto a graphene-covered Pt(111) was calculated:

$$\Delta G_{gr,n} = \Delta G_{f,n} - \Delta E_{gr/Pt} = \Delta G_{f,n} - \min_j \Delta E_{f,0,0,j} \quad \text{(S10)}$$

where $\Delta E_{f,0,0,j}$ is the most stable energy for graphene on Pt(111). We used this formation energy with respect to graphene-covered Pt(111) to calculate the change in binding energy for coverage n:

$$\Delta\Delta G_{gr,n} = \Delta G_{gr,n} - \Delta G_{Pt,n} \quad \text{(S11)}$$

S.2.2. Examples for thermodynamic methods

To illustrate the thermodynamic methods described above, we first consider a fictitious system in which two hydrogen atoms are adsorbed either on Pt(111) or on the interface of Pt(111) and the graphene overlayer. Our goal is to calculate the graphene effect $\Delta\Delta G_{gr,2}$ on Pt-H bonds in systems with 2/9 ML *H coverage. For the sake of clarity, we will first focus on hydrogenated Pt(111). First, we obtain the DFT energies for hydrogenated Pt(111) $U(PtH_{2,i})$ for various configurations *i* of *H. Each have an associated number of equivalent configurations W_{conf} as described in S.1.2. Assuming that the free energy of H_2(g) formation $G(H_2(g))$ is -7.00 eV and the DFT energy of the Pt(111) slab $U(Pt)$ is -200.00 eV, we obtain their individual formation energies $\Delta E_{f,n,i}$ as listed in Table S1. Using the number of configurations W_{conf}, and its associated TS_{conf} at T = 300 K as defined in S.1.2, we obtain the configurational entropy corrected formation energies $\Delta E_{f,n,i} - TS_{conf,n,i}$. One thing emanating from this calculation is that for the Pt calculations, *H configuration *i = 2* has a lower corrected formation energy than *i = 1*, despite its ΔE_f being more positive. The associated single configuration partition functions $z_{f,n,i}$, the total partition function for the set of configurational entropy corrected formation energies $Z_{f,n}$, and the resulting net probabilities $P_{f,n,i}$ are calculated as described in Equation S3, and are listed in Table S1. Moreover, the individual partition functions are used as parameters in Equation S5 to obtain a free energy term $\Delta E_{f,2}$ for the *H coverage based entirely on energetics and configurational entropy, also listed in Table S1 under $\Delta E_f - TS_{conf}$.

Table S1.

n*H (-)	Configuration	U (eV)	ΔE_f (eV)	W_{conf} (-)	ΔE_f-TS_{conf} (eV)	z (-)	Z (-)	P_f
**2**	***Total weighted***	-	-	-	**-1.28**	-	-	**1**
	**$PtH_{2,1}$**	-231.20	-1.20	9	-1.26	1.426×10^{21}	4.531×10^{21}	0.438
	**$PtH_{2,2}$**	-231.18	-1.18	27	-1.26	1.623×10^{21}	4.531×10^{21}	0.499
	**$PtH_{2,3}$**	-231.15	-1.15	3	-1.18	2.026×10^{20}	4.531×10^{21}	0.062

Example formation energy calculations excluding vibrational corrections.

No single configuration accounts for 50% of all possible observed configurations. However, the two most probable configurations combined (i = 1, 2) account for more than 50% of possible observed configurations, so they will be used for vibrational-energy calculations. Vibrational corrections $\Delta G_{vib,2,i}$ are added to the previously calculated $\Delta E_{f,2,i} - TS_{conf,2,i}$ to obtain new partition functions z_{vib}, cumulative partition function Z_{vib}, and probabilities $P_{vib,2,i}$ as described in Equation S4, which are used to obtain a weighted average vibrational correction $\Delta G_{vib,2}$. This correction is added to the previously calculated $\Delta E_{f,2}$ free-energy term to obtain the net free energy of formation for 2/9 ML *H on Pt(111): $\Delta G_{Pt,2}$, which in this example is -0.97 eV.

Subsequently, the corresponding graphene calculations are performed. Here, we assume a formation energy $U(\text{gr})$ of -23.00 eV for graphene. Both for 0 *H, which is graphene on otherwise pristine Pt(111), and for 2/9 ML *H, we calculate DFT energies $U(\text{grPtH}_{n,i,j})$ for various configurations *i* = 1, 2 of *H (if applicable) and *j* = A,B,C of graphene, as listed in Table S3. We use the energies described above to calculate formation energy $\Delta E_{f,n,i,j}$ by means of Equation S8, and, similarly to the Pt(111) *H configurations, correct these by TS_{conf} which are also listed in Table S3. In this dataset, each *H configuration *i* is combined with each graphene configuration *j*, and only the most stable graphene configurations for each *H configuration are used for vibrational analysis: $grPtH_{2,1,A}$ and $grPtH_{2,2,B}$. From their energies, configurational entropies and G_{vib}, the total $\Delta G_{f,2,i}$ for both *H configurations is calculated, and are combined into a cumulative free energy for the entire coverage, $\Delta G_{f,2}$, as illustrated in Table S3. For the entire coverage, $\Delta G_{gr,2}$ is calculated using Equation S9, using $grPtH_{0,0,C}$ as a reference. The resulting binding energy amounts to -0.33 eV. Finally, the difference $\Delta\Delta G_{gr}$ between the binding energy of *H on Pt(111), $\Delta G_{Pt,2}$, and the binding energy of *H on the interface of graphene and Pt(111), $\Delta G_{gr,2}$, is calculated, which is 0.65 eV in the example considered.

Table S2.

n*H (-)	Configuration	ΔE_f (eV)	ΔE_f-TS_{conf} (eV)	G_{vib} (eV)	z_{vib} (-)	P_{vib} (eV)	ΔG_{Pt} (eV)
**2**	***Total weighted***	-	**-1.28**	**0.31**	-	**1**	**-0.97**
	**$PtH_{2,1}$**	-1.20	-1.26	0.32	5.853×10^{15}	0.29	-0.94
	**$PtH_{2,2}$**	-1.18	-1.26	0.30	1.447×10^{16}	0.71	-0.96
	**$PtH_{2,3}$**	-1.15	-1.18	-		-	-

Example vibrational energy and total free energy calculations on pristine Pt(111).

Table S3.

n*H (-)	Configuration	U (eV)	ΔE_f (eV)	W_{conf} (-)	$\Delta E_f - TS_{conf}$ (eV)	G_{vib} (eV)	G_f (eV)	ΔG_{gr} (eV)	$\Delta\Delta G_{gr}$ (eV)
**0**	**$grPtH_{0,0,A}$**	-223.00	0.000	-	-	-	-	-	-
	**$grPtH_{0,0,B}$**	-223.48	-0.475	-	-	-	-	-	-
	**$grPtH_{0,0,C}$**	-223.50	-0.500	-	-	-	-	-	-
**2**	***Total coverage***	**-**	**-**	**-**	**-**	**-**	**-0.83**	**-0.33**	**0.65**
	**$grPtH_{2,1,A}$**	-224.60	-1.10	9	-1.16	0.34	-0.82	-0.32	-
	**$grPtH_{2,1,B}$**	-224.52	-1.02	9	-1.08	-	-	-	-
	**$grPtH_{2,1,C}$**	-224.56	-1.06	9	-1.11	-	-	-	-
	**$grPtH_{2,2,A}$**	-224.55	-1.05	18	-1.12	-	-	-	-
	**$grPtH_{2,2,B}$**	-224.55	-1.05	18	-1.13	0.33	-0.80	-0.30	-
	**$grPtH_{2,2,C}$**	-224.54	-1.04	18	-1.12	-	-	-	-

Example vibrational energy and total free energy calculations.

S.3. Thermodynamic data

Table S4.

n*H (-)	Configuration[a]	Functional	ΔE_f (eV)	$\Delta E_f - TS_{conf}$ (eV)	$\Delta E_{f,top}$ (eV)	P_f (-)	G_{vib} (eV)	P_{vib} (-)	ΔG_{Pt} (eV)
**1**	***Total weighted***	**PBE**	-	**-0.49**	-	1.00	**0.13**	1.00	**-0.35**
		**PBE-D3**	-	**-0.54**	-	1.00	**0.15**	1.00	**-0.39**
		**optPBE-vdW**	-	**-0.38**	-	1.00	**0.14**	1.00	**-0.24**
	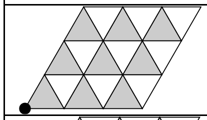	PBE	-0.35	-0.40	-	0.03	-	-	-
		PBE-D3	-0.42	-0.47	-	0.07	-	-	-
		optPBE-vdW	-0.29	-0.34	-	0.36	0.17	0.19	-
	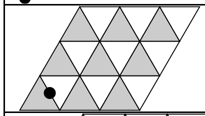	PBE	-0.37	-0.45	-	0.22	-	-	-
		PBE-D3	-0.41	-0.49	-	0.19	-	-	-
		optPBE-vdW	-0.25	-0.34	-	0.26	-	-	-
	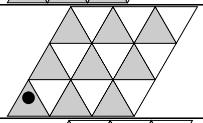	PBE	-0.38	-0.43	-	0.10	0.14	0.04	-
		PBE-D3	-0.42	-0.48	-	0.10	0.14	0.17	-
		optPBE-vdW	-0.23	-0.29	-	0.04	-	-	-
	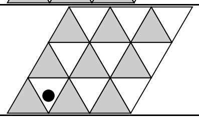	PBE	-0.43	-0.48	-	0.65	0.14	0.96	-
		PBE-D3	-0.47	-0.53	-	0.64	0.15	0.83	-
		optPBE-vdW	-0.29	-0.34	-	0.34	0.14	0.81	-
**2**	***Total weighted***	**PBE**	-	**-0.88**	-	1.00	**0.29**	1.00	**-0.59**
		**PBE-D3**	-	**-0.96**	-	1.00	**0.30**	1.00	**-0.67**
		**optPBE-vdW**	-	**-0.65**	-	1.00	**0.29**	1.00	**-0.37**
	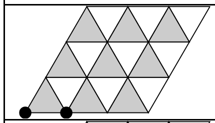	PBE	-	-	-	-	-	-	-
		PBE-D3	-	-	-	-	-	-	-
		optPBE-vdW	-0.55	-0.63	-	0.18	-	-	-
	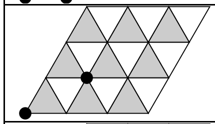	PBE	-	-	-	-	-	-	-
		PBE-D3	-	-	-	-	-	-	-
		optPBE-vdW	-0.55	-0.61	-	0.08	-	-	-
	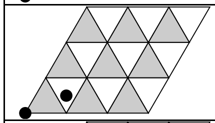	PBE	-	-	-	-	-	-	-
		PBE-D3	-	-	-	-	-	-	-
		optPBE-vdW	-0.55	-0.64	-	0.22	0.31	0.18	-
	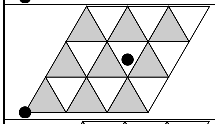	PBE	-	-	-	-	-	-	-
		PBE-D3	-	-	-	-	-	-	-
		optPBE-vdW	-0.55	-0.64	-	0.22	0.31	0.20	-
	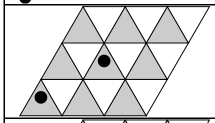	PBE	-0.72	-0.80	-	0.02	0.29	1.00	-
		PBE-D3	-0.80	-0.88	-	0.02	0.30	1.00	-
		optPBE-vdW	-	-	-	-	-	-	-
	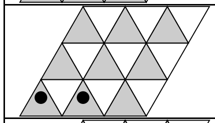	PBE	-0.71	-0.76	-	0.00	-	-	-
		PBE-D3	-0.79	-0.85	-	0.00	-	-	-
		optPBE-vdW	-	-	-	-	-	-	-
	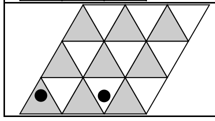	PBE	-0.76	-0.85	-	0.08	-	-	-
		PBE-D3	-0.84	-0.93	-	0.09	-	-	-
		optPBE-vdW	-	-	-	-	-	-	-

n*H (-)	Configuration[a]	Functional	ΔE_f (eV)	$\Delta E_f - TS_{conf}$ (eV)	$\Delta E_{f,top}$ (eV)	P_f (-)	G_{vib} (eV)	P_{vib} (-)	ΔG_{Pt} (eV)
**2**	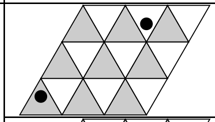	PBE	-0.75	-0.84	-	0.06	-	-	-
		PBE-D3	-0.83	-0.92	-	0.06	-	-	-
		optPBE-vdW	-	-	-	-	-	-	-
	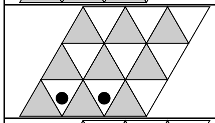	PBE	-0.82	-0.90	-	0.68	-	-	-
		PBE-D3	-0.90	-0.98	-	0.66	-	-	-
		optPBE-vdW	-0.55	-0.63	-	0.18	0.28	0.63	-
	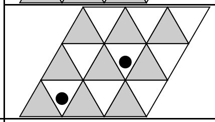	PBE	-0.81	-0.86	-	0.16	-	-	-
		PBE-D3	-0.89	-0.95	-	0.17	-	-	-
		optPBE-vdW	-0.54	-0.62	-	0.12	-	-	-
**3**[b]	***Total weighted***	**PBE**	-	**-1.24**	-	1.00	-	-	**-0.81**
		**PBE-D3**	-	**-1.35**	-	1.00	-	-	**-0.91**
		**optPBE-vdW**	-	**-0.91**	-	1.00	-	-	**-0.48**
**4**[b]	***Total weighted***	**PBE**	-	**-1.61**	-	1.00	-	-	**-1.03**
		**PBE-D3**	-	**-1.76**	-	1.00	-	-	**-1.17**
		**optPBE-vdW**	-	**-1.11**	-	1.00	-	-	**-0.53**
**5**[b]	***Total weighted***	**PBE**	-	**-1.91**	-	1.00	-	-	**-1.18**
		**PBE-D3**	-	**-2.09**	-	1.00	-	-	**-1.35**
		**optPBE-vdW**	-	**-1.29**	-	1.00	-	-	**-0.57**
**6**[b]	***Total weighted***	**PBE**		**-2.18**		1.00	-	-	**-1.31**
		**PBE-D3**		**-2.40**		1.00	-	-	**-1.52**
		**optPBE-vdW**		**-1.43**		1.00	-	-	**-0.57**
**7**	***Total weighted***	**PBE**		**-2.43**	-	1.00	**1.05**	1.00	**-1.41**
		**PBE-D3**		**-2.70**	-	1.00	**1.06**	1.00	**-1.67**
		**optPBE-vdW**		**-1.52**	-	1.00	**0.99**	1.00	**-0.52**
	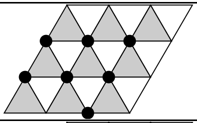	PBE	**-**	**-**	-	**-**	-	-	-
		PBE-D3	**-**	**-**	-	**-**	-	-	-
		optPBE-vdW[c]	-1.61	-1.69	-	0.00	1.29	**0.00**	-
	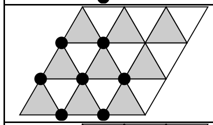	PBE			-		-	-	-
		PBE-D3	**-**	**-**	-	**-**	-	-	-
		optPBE-vdW[c]	-1.61	-1.66	-	0.22	-	-	-
	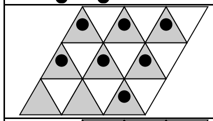	PBE	-1.99	-2.07	-	0.00	-	-	-
		PBE-D3	-2.27	-2.35	-	0.00	-	-	-
		optPBE-vdW	-	**-**	-	-	-	-	-
	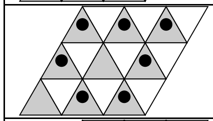	PBE	-1.97	-2.03	-	0.00	-	-	-
		PBE-D3	-2.25	-2.31	-	0.00	-	-	-
		optPBE-vdW	-	**-**	-	-	-	-	-
	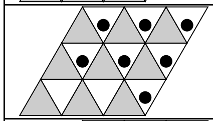	PBE	-2.34	-2.43	-	0.84	1.05	1.00	-
		PBE-D3	-2.61	-2.69	-	0.82	1.06	1.00	-
		optPBE-vdW[c]	-1.43	-1.52	-	0.00	0.99	**1.00**	-
	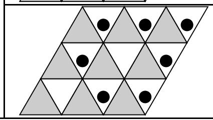	PBE	-2.33	-2.39	-	0.16	-	-	-
		PBE-D3	-2.60	-2.65	-	0.18	-	-	-
		optPBE-vdW[c]	-1.42	-1.48	-	0.00	-	-	-

n*H (-)	Configuration[a]	Functional	ΔE_f (eV)	$\Delta E_f - TS_{conf}$ (eV)	$\Delta E_{f,top}$ (eV)	P_f (-)	G_{vib} (eV)	P_{vib} (-)	ΔG_{Pt} (eV)
**8**	***Total weighted***	**PBE**	-	**-2.97**	-	1.00	**1.20**	1.00	**-1.50**
		**PBE-D3**	-	**-2.66**	-	1.00	**1.22**	1.00	**-1.79**
		**optPBE-vdW**	-	**-1.62**	-	1.00	**1.14**	1.00	**-0.47**
	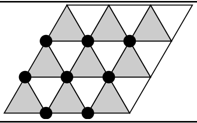	PBE	-	-	-	-	-	-	-
		PBE-D3	-	-	-	-	-	-	-
		optPBE-vdW[c]	-1.77	-1.83	-	0.00	1.48	0.00	
	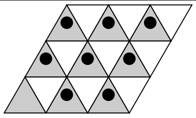	PBE	-2.19	-2.25	-	0.00	-	-	-
		PBE-D3	-2.51	-2.57	-	0.00	-	-	-
		optPBE-vdW[c]	-	-	-	-	-	-	-
	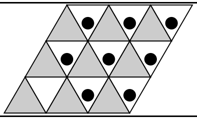	PBE	-2.61	-2.66	-	1.00	1.20	1.00	
		PBE-D3	-2.92	-2.97	-	1.00	1.22	1.00	
		optPBE-vdW[c]	-1.57	-1.62	-	1.00	1.14	1.00	-
**9**	***Total weighted***	**PBE**	-	**-2.87**	-	**1.00**	**1.22**	-	**-1.56**
		**PBE-D3**	-	**-3.21**	-	**1.00**	**1.39**	-	**-1.89**
		**optPBE-vdW[c]**	-	**-1.70**	-	**1.00**	**1.29**	-	**-0.41**
	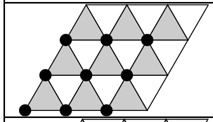	PBE	-2.33	-2.33	-	0.00	-	-	-
		PBE-D3	-3.03	-3.03	-	0.00	-	-	-
		optPBE-vdW[c]	-1.90	-1.90	-	0.00	1.68	0.00	-
	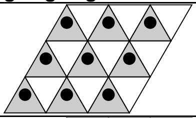	PBE	-2.38	-2.38	-	0.00	-	-	-
		PBE-D3	-2.73	-2.73	-	0.00	-	-	-
		optPBE-vdW[c]	-1.19	-1.19	-	0.00	-	-	-
	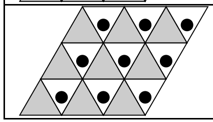	PBE	-2.87	-2.87	-	1.00	**1.22**	**1.00**	-
		PBE-D3	-3.21	-3.21	-	1.00	**1.39**	**1.00**	-
		optPBE-vdW[c]	-1.70	-1.70	-	1.00	**1.29**	**1.00**	-

Selected individual *H configurations and coverage thermodynamics on Pt(111).

a: Configurations are denoted as described in Figure S1.

b: Vibrational energies for this *H coverage are calculated using the average G_{vib} for 1, 2, 7, 8, and 9 *H atom coverages.

c: Probabilities for these configurations are based on $E_f - TS_{conf} - G_{vib}$.

Table S4.

n*H (-)	Configuration[a]	Functional	ΔE_f (eV)	$\Delta E_f - TS_{conf}$ (eV)	P_f (-)	G_{vib} (eV)	ΔG_{gr} (eV)	$\Delta\Delta G_{gr}$ (eV)
**0**	***Total weighted***	**PBE**	**0.07**	-	-	-	**0.00**	-
		**PBE-D3**	**-1.47**	-	-	-	**0.00**	-
		**optPBE-vdW**	**-1.65**	-	-	-	**0.00**	-
	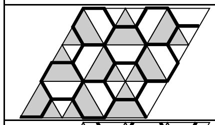	PBE	0.07	-	-	-	-	-
		PBE-D3	-1.46	-	-	-	-	-
		optPBE-vdW	-1.65	-	-	-	-	-
	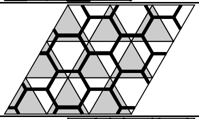	PBE	0.07	-	-	-	-	-
		PBE-D3	-1.47	-	-	-	-	-
		optPBE-vdW	-1.65	-	-	-	-	-
	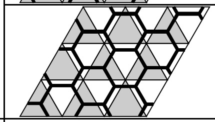	PBE	0.07	-	-	-	-	-
		PBE-D3	-1.46	-	-	-	-	-
		optPBE-vdW	-1.64	-	-	-	-	-
**1**	***Total weighted***	PBE	-	-	-	-	**-0.37**	**-0.02**
		PBE-D3	-	-	-	-	**-0.31**	**0.08**
		optPBE-vdW	-	-	-	-	**-0.17**	**0.07**
	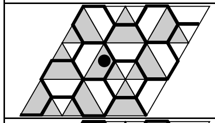	PBE	-0.12	-0.18	0.00	**-**		-
		PBE-D3	-1.69	-1.75	0.04	**0.15**	**-0.26**	-
		optPBE-vdW	-1.84	-1.90	0.03	**0.14**	**-0.12**	-
	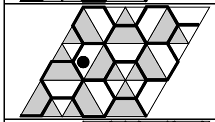	PBE	-0.24	-0.30	0.35			-
		PBE-D3	-1.74	-1.80	0.36	**0.16**	**-0.31**	-
		optPBE-vdW	-1.90	-1.96	0.35	**0.14**	**-0.17**	-
	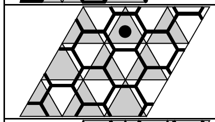	PBE	-0.19	-0.23	0.03	**0.14**	**-0.32**	-
		PBE-D3	-1.68	-1.73	0.02	-	-	-
		optPBE-vdW	-1.84	-1.89	0.02	-	-	-
	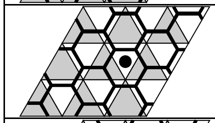	PBE	-0.24	-0.30	0.35	**0.15**	**-0.36**	-
		PBE-D3	-1.74	-1.80	0.28	-	-	-
		optPBE-vdW	-1.90	-1.96	0.32	-	-	-
	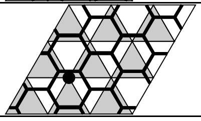	PBE	-0.13	-0.19	0.01	**0.17**	**-0.23**	-
		PBE-D3	-1.49	-1.54	0.00	-	-	-
		optPBE-vdW	-1.71	-1.77	0.00	-	-	-
**2**	***Total weighted***	PBE	**-**	**-**	**-**	**-**	**-0.62**	**-0.01**
		PBE-D3	**-**	**-**	**-**	**-**	**-0.55**	**0.06**
		optPBE-vdW	**-**	**-**	**-**	**-**	**-0.29**	**0.04**
	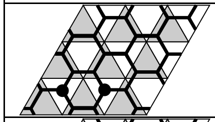	PBE	-0.75	-0.83	0.83	0.29	-0.61	-
		PBE-D3	-2.25	-2.34	0.80	0.32	-0.54	-
		optPBE-vdW	-2.13	-2.22	0.81	0.29	-0.28	-
	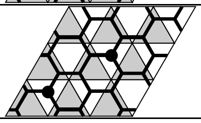	PBE	-0.73	-0.79	0.17	0.29	-0.57	-
		PBE-D3	-2.24	-2.30	0.20	0.32	-0.51	-
		optPBE-vdW	-2.12	-2.18	0.19	0.28	-0.25	-
**7**	***Total weighted***	PBE	-	-	-	-	**-1.26**	**0.16**
		PBE-D3	-	-	-	-	**-1.26**	**0.41**
		optPBE-vdW	-	-	-	-	**-0.33**	**0.18**

n*H (-)	Configuration[a]	Functional	ΔE_f (eV)	ΔE_f - TS_{conf} (eV)	P_f (-)	G_{vib} (eV)	ΔG_{gr} (eV)	$\Delta\Delta G_{gr}$ (eV)
	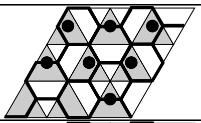	PBE	-1.84	-1.93	0.00	1.00	-1.00	-
		PBE-D3	-3.39	-3.48	0.00	1.07	-0.94	-
		optPBE-vdW	-	-	-	-	-	-
7	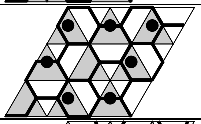	PBE	-1.83	-1.89	0.00	1.00	-0.96	-
		PBE-D3	-3.39	-3.44	0.00	1.06	-0.91	-
		optPBE-vdW	-	-	-	-	-	-
	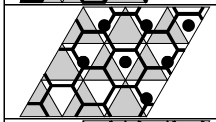	PBE	-	-	-	-	-	-
		PBE-D3	-3.75	-3.84	0.81	1.11	-1.25	-
		optPBE-vdW	-2.91	-2.99	0.54	1.01	-0.33	-
	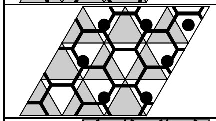	PBE	-	-	-	-	-	-
		PBE-D3	-3.74	-3.80	0.19	1.11	-1.22	-
		optPBE-vdW	-2.89	-2.95	0.10	1.02	-0.28	-
	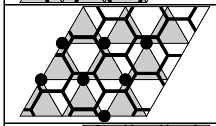	PBE	-	-	-	-	-	-
		PBE-D3	-	-	-	-	-	-
		optPBE-vdW	-2.89	-2.98	0.29	1.29	-0.05	-
	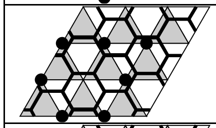	PBE	-	-	-	-	-	-
		PBE-D3	-	-	-	-	-	-
		optPBE-vdW	-2.89	-2.94	0.08	1.28	-0.01	-
	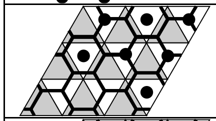	PBE	-2.19	-2.27	0.84	1.16	-1.18	-
		PBE-D3	-	-	-	-	-	-
		optPBE-vdW	-	-	-	-	-	-
	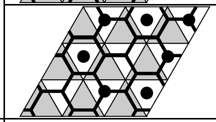	PBE	-2.17	-2.23	0.16	1.05	-1.26	-
		PBE-D3	-	-	-	-	-	-
		optPBE-vdW	-	-	-	-	-	-
8	***Total weighted***	PBE	-	-	-	-	**-1.36**	**0.02**
		PBE-D3	-	-	-	-	**-1.34**	**0.06**
		optPBE-vdW	-	-	-	-	**-0.27**	**0.03**
	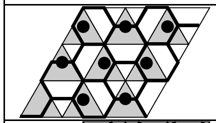	PBE	-2.03	-2.08	0.00	1.16	-1.00	-
		PBE-D3	-5.68	-5.74	0.00	1.23	-0.98	-
		optPBE-vdW	-	-	-	-	-	-
	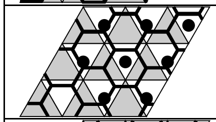	PBE	-	-	-	-	-	-
		PBE-D3	-	-	-	-	-	-
		optPBE-vdW	-3.06	-3.12	0.79	1.48	0.01	-
	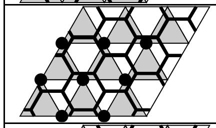	PBE	-	-	-	-	-	-
		PBE-D3	-6.10	-6.16	1.00	1.28	-1.34	-
		optPBE-vdW	-3.03	-3.09	0.21	1.17	-0.27	-
	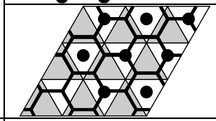	PBE	-2.45	-2.50	1.00	1.21	-1.36	-
		PBE-D3	-	-	-	-	-	-
		optPBE-vdW	-	-	-	-	-	-
9	***Total weighted***	PBE					**-1.45**	**0.01**
		PBE-D3					**-1.33**	**0.05**
		optPBE-vdW					**-0.23**	**0.01**

n*H (-)	Configuration[a]	Functional	ΔE_f (eV)	ΔE_f - TS_{conf} (eV)	P_f (-)	G_{vib} (eV)	ΔG_{gr} (eV)	$\Delta\Delta G_{gr}$ (eV)
**9**	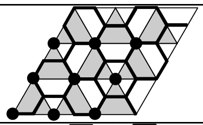	PBE	-	-	-	-	-	-
		PBE-D3	-	-	-	-	-	-
		optPBE-vdW	-3.21	-3.21	0.91	1.68	0.06	-
	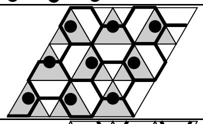	PBE	-2.21	-2.21	0.00	1.32	-1.02	-
		PBE-D3	-3.82	-3.82	0.00	1.45	-0.96	-
		optPBE-vdW	-	-	-	-	-	-
**9**	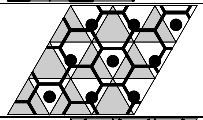	PBE	-2.65	-2.65	0.00	-	-	-
		PBE-D3	-4.31	-4.31	1.00	1.45	-1.45	-
		optPBE-vdW	-3.15	-3.15	0.09	1.32	-0.23	-
	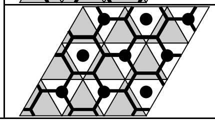	PBE	-2.69	-2.77	1.00	1.38	-1.44	-
		PBE-D3	-4.30	-4.30	0.00	-	-	-
		optPBE-vdW	-3.11	-3.11	0.00	-	-	-

Individual *H Configurations and Coverage Energies on Graphene/Pt(111).

a: Configurations are denoted as described in Figure S1.

S.4. Hydrogen atom diffusion and permeation

Table S6.

Surface	Site	E_{DFT} (eV)	G_{vib} (eV)	G (eV)	$\Delta G^{\ddagger}$ (eV)
**Pt(111)**	fcc	-231.29	0.15	-231.14	0.05
	bridge (TS)	-231.23	0.14	-231.09	-
	hcp	-231.24	0.14	-231.10	0.01
**Graphene/Pt(111)**	fcc	-454.82	0.16	-454.66	0.09
	bridge (TS)	-454.72	0.16	-454.56	-
	hcp	-454.76	0.15	-454.61	0.04

*H diffusion on platinum underneath graphene.

Table S7.

	ΔE$_f$ from Pt(111), graphene (eV)[b]		ΔE$_f$ from graphene/Pt(111) (eV)		
**Configuration**[a]	**-**	**+TS$_{conf}$**	**-**	**+ TS$_{conf}$**	**ΔE$_f$ – ΔG$_{gr,1}$**
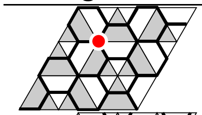	0.03	-0.06	0.15	1.27	1.59
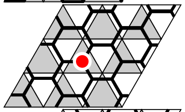	0.08	-0.01	0.14	1.32	1.64
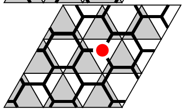	0.08	0.00	0.14	1.33	1.64

Hydrogenated graphene energies.

a Configurations illustrated as described in Figure S1, with graphene-binding H represented using a red circle.
b E$_f$ as described in Subsection S.1.2.

Fig. S2.

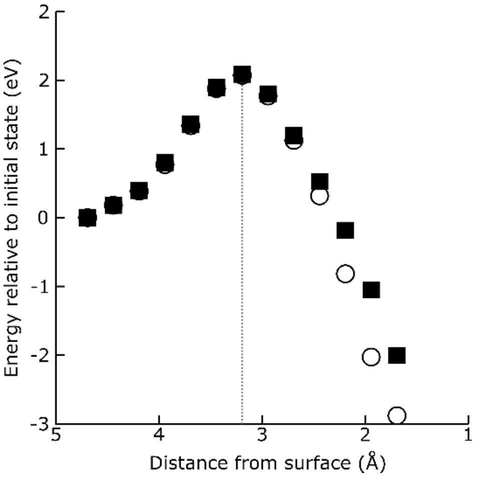

Hydrogen atom energies with respect to the barrier as a function of distance from the graphene layer. Solid squares and open circles represent the hydrogen atom diffusing through graphene onto a bridge site, and onto a top site, respectively.

Table S8.

Vacancy type	nH (-)	Configuration[a]	ΔE_f (eV)	ΔE_f-TS_{conf} (eV)
mono-vacancy	0	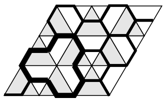	0.00	0.00
	1	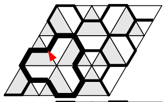	-0.40	-0.43
	2	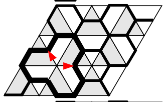	-0.05	-0.08
	2	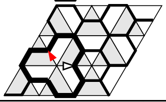	-0.65	-0.69
far divacancy	0	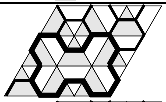	0.00	0.00
	1	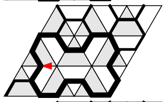	-1.55	-1.57
	1	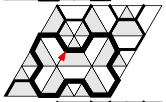	-0.27	-0.31
	2	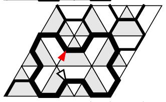	1.31	1.28
	2	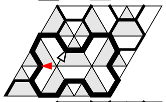	-1.78	-1.81
	2	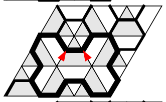	-2.04	-2.05
	2	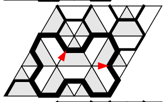	-1.54	-1.57
	2	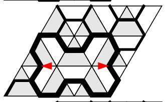	-2.40	-2.40
	3	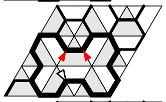	-1.71	-1.75
	3	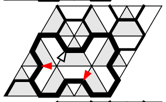	-1.67	-1.70
	3	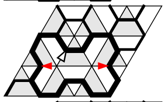	-2.35	-2.39
	3	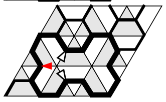	-1.58	-1.60

Vacancy type	nH (-)	Configuration[a]	ΔE_f (eV)	E_f-TS_{conf} (eV)
far divacancy	4	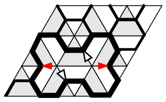	-2.17	-2.19
	4	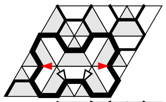	-2.14	-2.16
	4	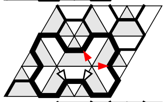	-1.53	-1.56
	4	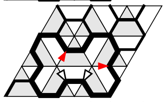	0.03	0.00
	4	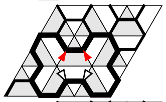	-1.16	-1.17
	5	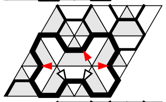	-1.91	-1.95
	5	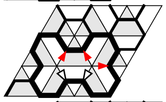	0.26	0.24
	6	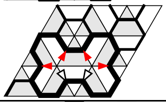	-1.55	-1.56
close divacancy	0	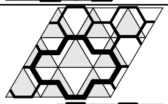	0.00	0.00
	1	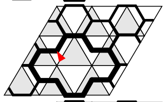	-1.08	-1.12
	2	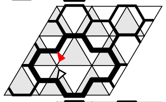	-2.13	-2.16
	2	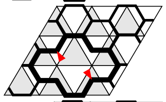	-1.27	-1.28
	2	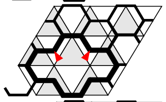	-2.96	-2.98
	3	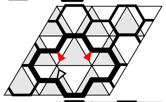	-3.94	-3.96
	4	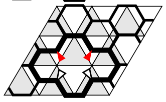	-4.94	-4.96

Energies of vacancy passivation.

a Configurations illustrated as described in Figure S1, with C-H bonds pointing towards the surface depicted as empty white triangles and C-H bonds pointing away from the surface depicted as solid red triangles.

Table S9.

Vacancy type	Configuration[a]	ΔE_f (eV)	ΔE_f-$\Delta E_{gr/Pt}$ (eV)[b]	Vacancy type	Configuration[a]	ΔE_f (eV)	ΔE_f-$\Delta E_{gr/Pt}$ (eV)[b]
far divacancy	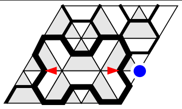	-0.02	0.39	close divacancy	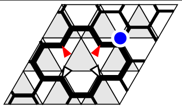	-0.18	0.24
	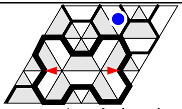	-0.18	0.23		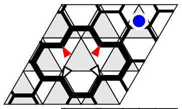	-0.33	0.09
	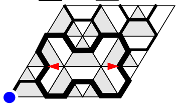	-0.18	0.24		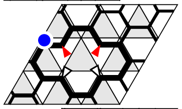	-0.18	0.23
	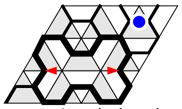	-0.09	0.32		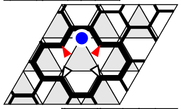	-0.37	0.05
	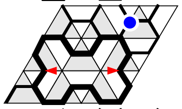	-0.19	0.23		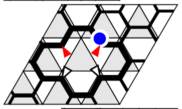	-0.33	0.08
	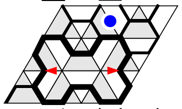	-0.17	0.24		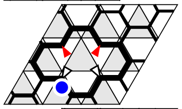	-0.05	0.36
	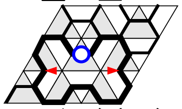	0.02	0.43		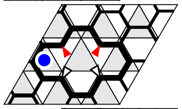	-0.14	0.27
	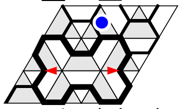	0.03	0.44		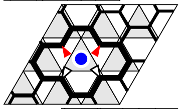	-0.06	0.35
	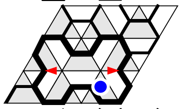	0.46	0.88		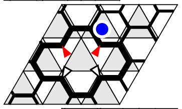	-0.35	0.07
	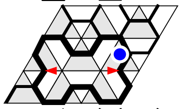	-0.15	0.27		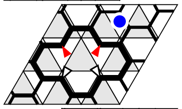	-0.38	0.03
	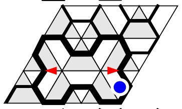	-0.19	0.23		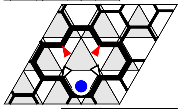	-0.06	0.36
	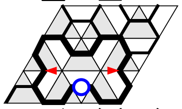	0.32	0.73		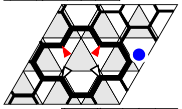	-0.34	0.08
	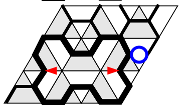	-0.24	0.18		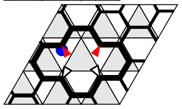	-0.38	0.03
close divacancy	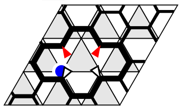	0.23	0.65		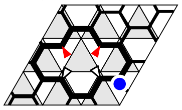	-0.21	0.20

Adsorption site binding energies for H under graphene vacancies.

a Configurations illustrated as described in Figure S1, with C-H bonds pointing towards the surface depicted as empty white triangles and C-H bonds pointing away from the surface depicted as solid red triangles.

b E_f compared to the lowest E_f for 1 *H between graphene and Pt(111).

S.5. vdW interactions and *H binding energies

In the following, we will compare the results of PBE and PBE-D3 at the level of binding energies, namely without including zero-point energy and entropy corrections, which tend to be relatively constant. The binding energies in kJ/mol per *H (ΔE_{nH}) are defined as:

$$\Delta E_{nH}^{PBE} = \left(E_{*nH} - E_{*} - \frac{n}{2}E_{H_2}\right)_{PBE}/n$$

(S12)

$$\Delta E_{nH}^{PBE-D3} = \left(E_{*nH} - E_{*} - \frac{n}{2}E_{H_2}\right)_{PBE-D3}/n$$

(S13))

where E_{*} is the total energy of the clean slab, E_{*nH} is the energy of the slab with *n* hydrogen atoms adsorbed, and E_{H_2} is the total energy of H_2. The difference in the adsorption energies is:

$$\Delta E_{nH}^{PBE} - \Delta E_{nH}^{PBE-D3} = \frac{1}{n}\left[\left(E_{*nH} - E_{*} - \frac{n}{2}E_{H_2}\right)_{PBE} - \left(E_{*nH} - E_{*} - \frac{n}{2}E_{H_2}\right)_{PBE-D3}\right]$$

(S14)

Reorganizing the terms, we have the following:

$$\Delta E_{nH}^{PBE} - \Delta E_{nH}^{PBE-D3} = \frac{1}{n}\left[(E_{*nH}^{PBE} - E_{*nH}^{PBE-D3}) - (E_{*}^{PBE} - E_{*}^{PBE-D3}) - \frac{n}{2}\left(E_{H_2}^{PBE} - E_{H_2}^{PBE-D3}\right)\right]$$(S15)

The difference between the total energies of the clean slabs ($E_{*}^{PBE} - E_{*}^{PBE-D3}$) is coverage-independent. In addition, the total energy difference between H_2 in PBE and PBE-D3 $\left(E_{H_2}^{PBE} - E_{H_2}^{PBE-D3}\right)$ is constant and negligible (2.8×10^{-3} kJ/mol). Thus, the differences in the adsorption energies of hydrogen come from the term ($E_{*nH}^{PBE} - E_{*nH}^{PBE-D3}$). As shown in Figure R1, for bare Pt(111) the binding-energy differences are positive and nearly constant as a function of the number of hydrogen atoms, which indicates that the term $(E_{*nH}^{PBE} - E_{*nH}^{PBE-D3})/n$ is coverage-independent and inclined towards PBE-D3. In other words, the strength of H-surface bonds is stabilized by dispersion interactions.

The situation is different for Pt(111) with a graphene overlayer, as the term $(E_{*nH}^{PBE} - E_{*nH}^{PBE-D3})/n$ changes linearly as a function of *H coverage. While that term is negative for low coverage, it becomes positive at high hydrogen coverage. This means that the graphene overlayers destabilize *H adsorption on Pt(111) by means of dispersion interactions at low coverages, although increasing hydrogen coverage strengthens those.

In brief, our main conclusions from Figure S3, which help rationalize the trends in Figure 7 in the main text are: (i) the difference in hydrogen adsorption energies between PBE and PBE-D3 for bare Pt(111) is approximately constant and inclined toward PBE-D3, (ii) dispersion interactions destabilize hydrogen adsorption on Pt(111) with a graphene overlayer at low coverages and slightly stabilize them at high coverages. Since the vdW contribution to the adsorption energy of *H on pristine Pt(111) is small and relatively constant, we can reason that vdW interactions between H atoms are mild towards their binding energies. Conversely, graphene interacts strongly with Pt(111): 1 ML graphene has a net vdW contribution (i.e. PBE-D3 – PBE) to the binding energy on Pt(111) of 142 kJ/mol (Table S4), whereas this contribution for 1 ML *H is 35 kJ/mol. Van der Waals interactions in general are inversely dependent on the distance between polarizable charge distributions. Repulsion from one single *H on Pt(111) forces graphene to move away from the surface, thereby weakening graphene-Pt(111) vdW interactions. Adding more *H causes some additional repulsion, yet these atoms bind at similar distances to the surface compared to low-coverage *H. Hence these additional atoms do not have as great an impact

on the binding energy of graphene, and net binding energies per *H trend closer to those on the non-graphene Pt(111) surface.

Fig. S3.

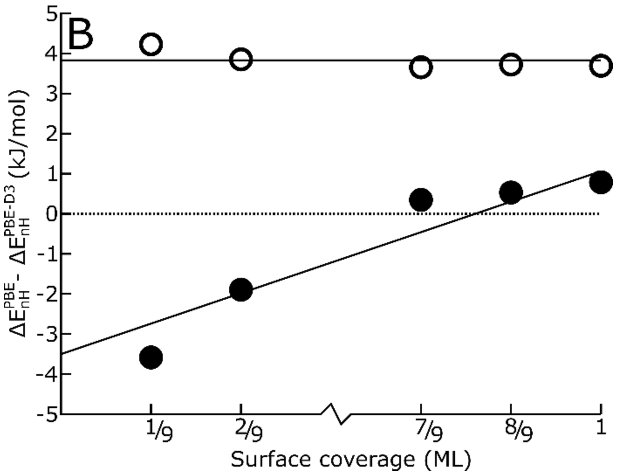

Differences in the adsorption energies of *H for PBE versus PBE-D3 as a function of the number of atoms adsorbed. Linear fits are included as guides for the eye. Filled: adsorption on bare Pt(111). Empty: adsorption on Pt(111) with a graphene overlayer.
